# Terminally Phosphorylated Triblock Polyethers Acting Both as Templates and Pore-Forming Agents for Surface Molecular Imprinting of Monoliths Targeting Phosphopeptides

**DOI:** 10.1021/acsomega.3c00007

**Published:** 2023-02-20

**Authors:** Chau Minh Huynh, Ignacio Arribas Díez, Hien Kim Le Thi, Ole N. Jensen, Börje Sellergren, Knut Irgum

**Affiliations:** † Department of Chemistry, Umeå University, S-901 87 Umeå, Sweden; ‡ Department of Biochemistry & Molecular Biology and VILLUM Center for Bioanalytical Sciences, University of Southern Denmark, Campusvej 55, DK-5230 Odense M, Denmark; § Faculty of Health and Society, Department of Biomedical Science, Malmö University, S-205 06 Malmö, Sweden

## Abstract

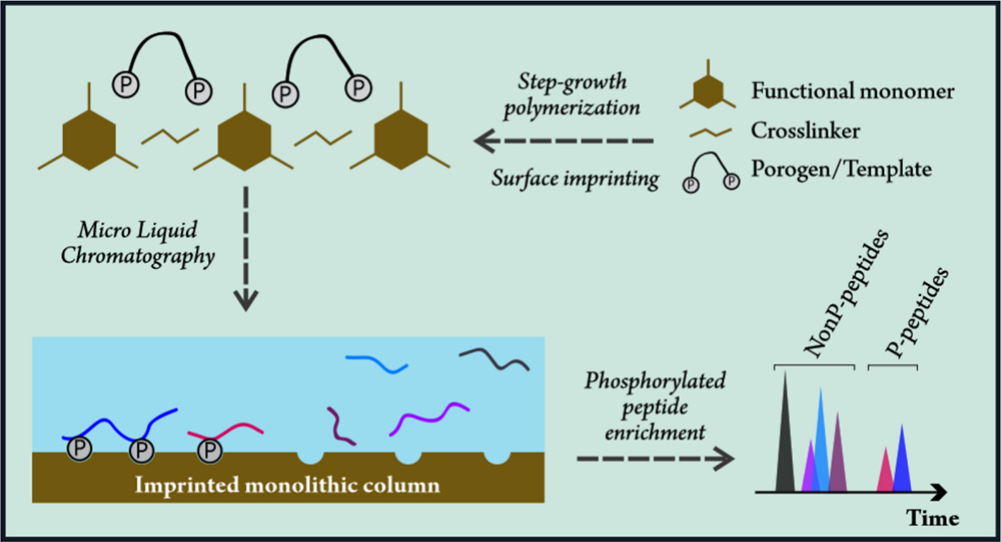

The novel process reported here described the manufacture of monolithic molecularly imprinted polymers (MIPs) using a terminally functionalized block copolymer as the imprinting template and pore-forming agent. The MIPs were prepared through a step-growth polymerization process using a melamine–formaldehyde precondensate in a biphasic solvent system. Despite having a relatively low imprinting factor, the use of MIP monolith in liquid chromatography demonstrated the ability to selectively target desired analytes. An MIP capillary column was able to separate monophosphorylated peptides from a tryptic digest of bovine serum albumin. Multivariate data analysis and modeling of the phosphorylated and nonphosphorylated peptide retention times revealed that the number of phosphorylations was the strongest retention contributor for peptide retention on the monolithic MIP capillary column.

## Introduction

Molecularly imprinted polymers (MIPs) rely on the formation of macromolecular structures which are intended to imitate the selective interactions of natural recognition entities in various applications, such as enrichment, sensing, and disease diagnosis.^1−6^ This synthetic scheme of establishing interactive surfaces with high binding strength and selectivity has several advantages compared to conventional antibodies, namely, better stability, lower production cost, and no need to use live animals or cell lines.^7^ The first preparation methods for MIPs were based on covalent bulk imprinting, with pioneering studies by Wulff et al.^8^ and Takagishi and Klotz.^9^ In the 1980s, Mosbach et al.^2,10−12^ introduced a noncovalent imprinting concept, which is prevalent in current research and mimics the antibody–antigen interaction mechanism.^13^ The basic bulk imprinting concept is based on the preparation of an organic polymer scaffold in the presence of *template*, intended to establish appropriate interactions between functional groups of the targeted compound (or an analog), and one or more functional monomer(s) selected to offer specific interactions with complementary functional groups of the template.^14^ During polymerization, the template and the functional monomers will rearrange in the reaction cocktail as the polymer is formed to reach spatial arrangements with minimal internal energy. A rigid and porous structure is meanwhile generated by added crosslinkers and porogens, respectively.^15^ When the polymerization is completed, the template is removed. In favorable cases, this leads to binding sites which can selectively trap the template or its analogs.

However, a critical drawback of traditional MIP synthesis is that the target itself, or a compound with high structural similarity, must be added in relatively large amounts to act as a template in the imprinting step.^16^ Complete removal of the template from the finished material can prove difficult, especially if the MIPs are prepared by bulk polymerization.^17^ MIPs prepared by this process are also hampered by characteristic tailing peaks on elution, due to diversity in binding site topographies and associated binding activities, caused by crushing of the bulk polymer to expose its imprinted sites.^18^ Therefore, other approaches, namely, oriented surface imprinting, epitope imprinting, and dual template docking oriented molecular imprinting (DTD-OMI) have been developed. In oriented surface printing, the removal of the template will result in specific binding sites only on the polymer surface,^19,20^ providing better access for the target molecule than bulk imprinting.^21−24^ In epitope printing, only a small portion or fragment of the target molecule (an epitope) is used as the template to create an imprint for the target,^21,25^ whereby more specific and strong interactions can be obtained. This can create imprints that recognize templates, peptides, or even whole proteins.^25^ The DTD-OMI technique involves the use of two templates, a functional template that directly interacts with the monomer to form the imprinted pocket and the other is a “dummy” template that helps orient the functional template in the desired position which provides higher selectivity and binding capacity compared to the bulk approach.^26^

In this work, we have chosen to prepare MIPs in the capillary monolith format by a cross-linking step-growth polymerization of a melamine-formaldehyde (MF) precondensate in a partly aqueous biphasic system with acetonitrile as the diluent^27^ using a terminally phosphorylated amphiphilic triblock polyether surfactant to play the combined roles of a selectivity-promoting template and a pore-forming agent. The underpinning idea was that phase separation which the diluent/porogen system will undergo by polymerization should promote a preferential orientation of the terminal phosphate groups in the hydrophilic outer blocks of the mesoporogen/template toward the aqueous MF precondensate phase where a cross-linking step-growth polymerization takes place, whereas the hydrophobic center block is expected to assist in the pore-forming process by preferential association with the acetonitrile diluent. We hence envisaged that this surface/epitope imprinting scheme could establish generic phospho-specific surface interaction sites of similar strength using a reduced amount of templates that should be easily removable after the imprinting is completed.

## Materials and Methods

### Reagents and Materials

The monomers used were 1, 3, 5-triazine-2, 4, 6-triamine (melamine, 99%) from Merck, Darmstadt, Germany, and polyoxymethylene (paraformaldehyde, extra pure) from BDH, Poole, UK. Acetonitrile (ACN, p.a.), formic acid (FA, 98–100%), phosphoryl trichloride (POCl_3_; 99%), α-hydro-ω-hydroxy-poly[oxy(1-methylethylene)] [PPG4000; poly(propylene oxide), 4000 Da], iodoacetamide (IAA), 1, 4-dithiothreitol (DTT), triethylammonium bicarbonate (TEAB) buffer (1 M), deuterium oxide (D_2_O, 99.9% atom-% D), and deuterated acetonitrile (CD_3_CN, >99.8 atom-% D) were from Merck. Trifluoroacetic acid (TFA) was a product of VWR Chemicals (Radnor, PA, USA). The methanol used in the Soxhlet extraction was of analytical grade from Prolabo, obtained from VWR. Tetrahydrofuran (THF, 99.5% anhydrous, < 0.005% H_2_O dried over molecular sieves, stabilized with 2, 6-di-*tert*-butyl-4-methylphenol) was from Scharlau (Barcelona, Spain). Phenylphosphoric acid (PPA) was a product of TCI (Geel, Belgium). The amphiphilic triblock copolymers Pluronic L61, L121, P123, and F127, α, ω-hydroxy-poly(oxyethylene)-*block*-poly[oxy(1-methylethylene)]-*block*-poly(oxyethylene) of varying overall molecular weights and block ratios were gifts from BASF (Ludwigshafen, Germany). Dialysis tubing (MWCO 1000 Da) was from Spectrum Laboratories (New Brunswick, NJ, USA). The GADDYYTAR peptides (non- and mono-phosphorylated on tyrosine) were custom-synthesized by LifeTein (Somerset, NJ, USA), and trypsin was from Promega (Madison, WI, USA). All chemicals were used as received, unless otherwise noted. Water used was prepared by Milli-Q or Ultra-Q equipment from Merck Millipore (Bedford, MA, USA).

### Preparation of Terminally Phosphorylated Triblock Polyether

**Figure 1:**
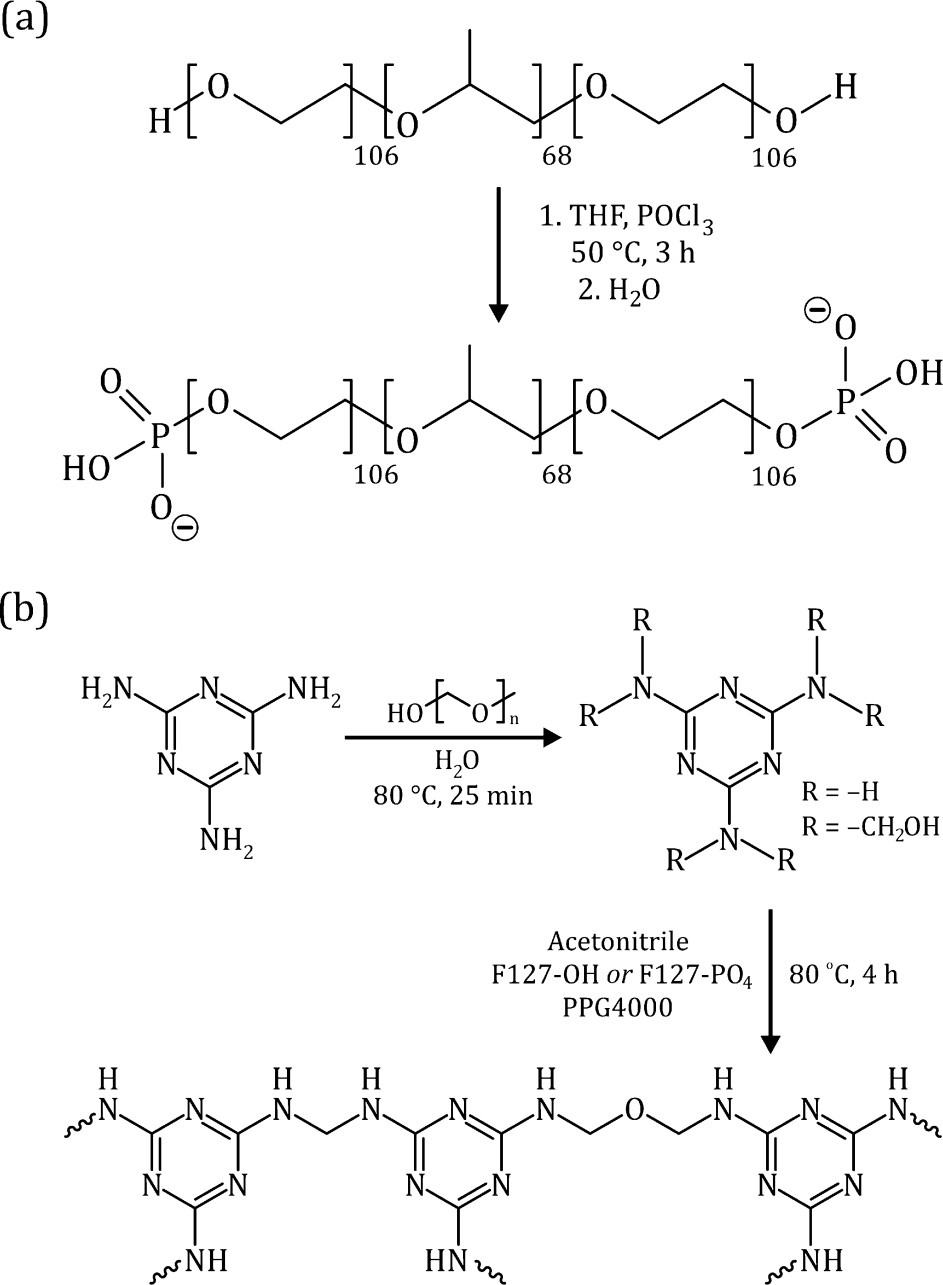
Synthesis routes (a) to the terminally phosphorylated triblock polyether, showing the two-step one-pot procedure consisting of phosphoesterification followed by hydrolysis, and (b) to the melamine-formaldehyde monoliths in two steps; formation of precondensate and polycondensation.

The α, ω-hydroxy-terminated triblock copolymers Pluronic L61, L121, P123, and F127 (0.25 mmol) were dissolved in separate 20.0 mL aliquots of dry THF at room temperature. The phosphorylation reagent, phosphoryl trichloride (0.45 mL; 4.8 mmol), was thereafter added dropwise to each Pluronic solution over a period of ≈5 min with magnetic stirring, after which the mixtures were heated to 50 °C and maintained for 3 h. The reactions were then quenched with water (1 mL), followed by the removal of solvents by rotary evaporation at 40 °C under vacuum (Figure 1a). The crude residues, which were colorless and somewhat viscous, were diluted by water (10 mL) and purified by dialysis against water for 72 h, with a change to fresh water every 12 h. The dialyzed products were finally dried by lyophilization. These phosphorylated Pluronics are referred to by their numerical designations with a −PO_4_ suffix.

### Pretreatment of Fused Silica Capillaries

Prior to synthesizing the monolithic packing, the polyimide-coated fused-silica capillaries (100 μm i.d., 375 μm o.d.) from Polymicro Technologies (Phoenix, AZ, USA) were first treated by an adapted etching protocol^28^ at room temperature comprising a rinse with methanol followed by deionized water, then etching with aqueous 1 M NaOH for 2 h followed by a rinse with deionized water until neutral. This was followed by flushing with 1 M aqueous HCl for 20 min, deionized water until neutral, methanol for 1 h, and drying by a flow of nitrogen at room temperature for 12 h. These etched capillaries were subsequently modified to produce three different activated capillaries (Figure S1; prefix “S” refers to the Supporting Information), first by 10% (v/v) (3-aminopropyl)triethoxysilane (APTES) in toluene at 60 °C overnight, followed by washing with water and methanol and drying as in the final step of the above etching procedure, yielding capillaries **C1**. Dried C1 capillaries were subsequently reacted according to two different schemes; capillaries **C2** were prepared by filling C1 with 10% (v/v) aqueous formaldehyde and reacting at 80 °C for 4 h, followed by washing and drying as above; capillaries **C3** were filled with the MF precondensate (see below) diluted 1 + 9 (v/v) with water and reacted at 80 °C for 4 h, followed by washing and drying as above. Activated capillaries were stored in a desiccator for use in the polymerization of MF monolithic columns.

### Preparation of Melamine–Formaldehyde Precondensate

Paraformaldehyde (1.00 g) and melamine (1.43 g) (molar ratio of formaldehyde–melamine 2.94:1) were added to 4.00 g of water at room temperature in a 10 mL glass vial provided with a magnetic stir bar. It was directly thereafter sealed by a PTFE-lined silicone septum, placed in an oil bath at 80 °C, and heated under stirring until the solution appeared clear after ≈25 min. This MF precondensate was then allowed to cool to room temperature and stored for a maximum of 4 h until the next steps.

### Preparation of the Mixed Porogen Solution

Co-porogen (PPG4000, 60.0 mg) and 540 mg of mesoporogen, which also served a dual role as a template for the MIP (F127-OH for NIP and F127-PO_4_ for MIP), were added to 6.00 mL of acetonitrile in 10 mL glass vials and capped with PTFE-lined silicone septa. The solutions were thereafter sonicated at room temperature for 5 min in an Emmi 30 Eco ultrasonic bath from EMAG Technologies (Walldorf, Germany) to ensure homogeneity, stored under ambient conditions, and given 5 min sonication prior to use.

### Preparation of Molecularly Imprinted Melamine–Formaldehyde Capillary Monoliths

The MF precondensate prepared as described above was used to synthesize both imprinted (MIPs) and nonimprinted (NIPs) monolithic MF polymers by the one-pot synthesis route shown in Figure 1b. In this procedure, the MF precondensate (780 μL) was mixed with NIP or MIP porogen solution (600 μL) and formic acid (35 μL) in a 2 mL GC glass vial, then ultrasonically agitated as above to prepare homogeneous solutions. The sonicated vials were capped with PTFE-lined septa, shaken vigorously, and given an additional 30 s ultrasonic treatment. Pretreated capillaries **C3** cut into 50 cm long pieces were then filled with the MF precursor solution by inserting one end through the pre-pierced septa of the precursor-containing vial and the other end likewise into an empty septum-capped vial. Slight N_2_ overpressure applied to the vial containing the precursor solutions caused a slow flow, keeping the capillary inlet submerged so that gas bubbles did not enter into the capillary. After several drops of precursor solution had exited from the capillary outlet, N_2_ pressure was released by piercing the septum of the pressurized vial by a hypodermic needle. The filled capillary was thereafter withdrawn from both vials and capped by piercing the ends into silicone GC septa. Both the capped capillaries and the left-over solutions in the source vials were transferred to a Binder ED53 convective oven (Tuttlingen, Germany) where polymerization took place under static conditions for 4 h at 80 °C. The capillaries were thereafter allowed to cool to ambient temperature.

The polymerized capillaries were detached from the septa and cut into monolith columns of 70 mm length. The glass vials were disintegrated with minimal force to render the bulk monolithic materials formed therein as intact as possible, then parting it into roughly cubiform pieces with 2–3 mm sides, which were transferred to cellulose extraction thimbles. Unreacted precondensate, mesoporogen/template, co-porogen, solvents, and reaction by-products were flushed from the capillaries by pumping with methanol for 24 h at 20 μL/min. The parted bulk monoliths were Soxhlet-extracted for 24 h with methanol. Cleaning of the capillary monoliths was followed by flushing with at least ten column volumes of 70:30% (v/v) acetonitrile/water, which was also used to store the capillary MIP and NIP monoliths in a refrigerator at +4 °C. The Soxhlet-extracted bulk monolithic materials were finally dried under reduced pressure (≈100 Pa) in a Gallenkamp (Loughborough, UK) vacuum oven at 40 °C overnight, followed by crushing and sieving (37–74 μm) prior to further characterization.

### Characterization and Evaluation

Experimental details of the characterization and evaluation procedures are provided in the Supporting Information.

## Results and Discussion

### Preparation of Terminally Phosphorylated Triblock Polyethers

In previous work with the melamine-formaldehyde monomer system,^27^ we have used a porogen combination consisting of Pluronic L121, an amphiphilic ABA triblock polyether surfactant (poloxamer) [poly(ethylene oxide)-*block*-poly(propylene oxide)-*block*-poly(ethylene oxide)], together with poly(ethylene oxide) or poly(propylene oxide) of varying molecular weight to control the formation of meso- and through-pores in monolithic materials based on the step-growth polymerization of a methylolated melamine precondensate. This spurred the idea to the present investigation, where our hypothesis was that a poloxamer/polyether co-porogen system could be used to produce a surface imprinted monolithic material with a bimodal pore structure and selectivity against peptides with *O*-phosphorylated amino acids, provided the hydroxyl terminals of the poloxamer were converted into phosphate groups, hence acting both as pore-directing elements and imprinting templates. In order to test this hypothesis, we first set out to prepare terminally phosphorylated triblock polyethers via a two-step procedure (Figure 1a) comprising (i) phosphoesterification of the bis-hydroxy-terminated triblock polyether by phosphoryl trichloride in dry THF and (ii) hydrolysis of the intermediate dichlorophosphate terminals to form phosphate groups. This was followed by dialysis against water and lyophilization to produce dry products free from water-soluble by-products of low molecular weight.

The first synthesis attempts were based on Pluronics L61, L121, P123, and F127, where the first two differ in the molecular weights of the poly(propylene oxide) blocks (1, 750 and 4, 000, respectively) but have identical mass ratios of the distal poly(ethylene oxide) blocks to the central poly(propylene oxide) blocks (10% of the total polymer mass). Pluronics P123 and F127 share the same length of the poly(propylene oxide) segment as Pluronic L121 (≈68 units), but the distal poly(ethylene oxide) blocks are extended to account for 30 and 70%, respectively, of their total masses (see Table S1). This resulted in products that produced quite different ^31^P NMR spectra. The ^31^P signals from L61-PO_4_ and F127-PO_4_ were triplets due to coupling with adjacent hydrogens and narrow singlets in decoupling mode, whereas the ^31^P signals from L121–PO_4_ and P123–PO_4_ were significantly broader and not clear of the hydrogen coupling triplet signals, suggesting bulky structures (Figure S2). Phosphorylation of L121 and P123 hence seemed to result in partial formation of phosphonate bridges, whereas L61-PO_4_ and F127-PO_4_ appeared to be essentially monomeric with phosphate terminals appropriate for the aims of this work. Of these two, we chose F127-PO_4_, nominally EO_106_PO_68_EO_106,_^29^ due to its higher MW of ≈13,330 and thereby an expected stronger structure-directing ability.

Parallel characterization of the as-received hydroxy-terminated Pluronic F127-OH and the phosphorylated Pluronic F127-PO_4_ by Fourier-transform infrared spectroscopy (FT-IR; Figure S3) and nuclear magnetic resonance (Figure S4) verified that the Pluronic F127-PO_4_ was an essentially pure phosphorylated product. The FT-IR peaks due to the F127 polymeric backbones seen at ≈2890 cm^–1^ in both spectra are associated with the C–H stretching vibrations of the methylene and methyl groups, the methylene C–H bending vibration is seen at 1465 cm^–1^, and the C–O–C stretching vibrations of the ether groups are at 1240, 1282, and 1344 cm^–1^. The Hotelling’s *T*^2^ plot shown below the stacked FT-IR spectra of F127-OH and F127-PO_4_ identifies the wavenumber ranges where the spectra differed the most. Significant differences are the O–H signal of F127-OH centered at 3504 cm^–1^, which disappeared in F127-PO_4_ and was replaced by the phosphorylated terminals in F127-PO_4_ evident in significant stretching vibrations from the phosphate groups at 962 cm^–1^ for P–O–C and 1114 cm^–1^ for P=O, verifying that the terminally phosphorylated triblock polyether was produced in high yields. The apparently significant change in the C–H stretching signals of the polymer backbone at around 2890 cm^–1^ is likely an artifact caused by the default centering and scaling procedure of SIMCA (see the Supporting Information).

To further confirm that the triblock polyether terminals had been functionalized into phosphate groups, ^1^H, ^13^C, and ^31^P NMR spectra were recorded for both Pluronic F127-OH and F127-PO_4_. Since the major part of the investigated samples consisted of identical triblock EO-PO-EO polyethers, the ^1^H, and ^13^C NMR spectra feature similar signals, in terms of chemical shift and integration. The ^13^C signals of methyl, methanetriyl, and methylene carbons in the PPO and PEO segments appeared at 17.44, 76.24, 73.55, and 70.82 ppm. Peaks at 1.05 ppm in the ^1^H NMR spectra were attributed to the methyl (−C**H**_3_) group protons of PPO and assigned normalized peak areas of 3.0. Proton signals from methanetriyl (−C**H**−) in the PPO segments and methylene (−C**H**_2_−) in both the PEO and PPO segments were located at chemical shifts of 3.40 ppm and 3.56 ppm, with combined normalized peak areas of 16.87 for F127-OH and 16.65 for F127-PO_4_. PEO segment weight percentages of F127-OH and F127-PO_4_, determined by Eq. S3 based on the proton NMR signal integrals, were 72.5 and 72.1%, respectively, which are well in agreement with the nominal 70% of EO in Pluronic F127.^30^ Moreover, the mesoporogen phosphorylated to act also as the template was evaluated by ^31^P NMR (Figure S5), where signals appeared only in the spectrum of F127-PO_4_, as expected. The singlet signal of ^31^P decoupling with ^1^H and the triplet signal with *J* = 8.1 Hz in coupling mode are consistent with a three-bond phosphorous–proton coupling.^31^ This further verified the presence of the terminal phosphate groups (Figure 1a).

In order to assess the feasibility of using F127-PO_4_/PPG4000 as a combined mesoporogen, co-porogen, and template for surface imprinting of phosphate groups, we determined the hydrodynamic size, ζ-potential, and the polydispersity index of micellar entities and aggregates in the co-porogen and/or mesoporogen solvent mixtures. Briefly, F127-OH and F127-PO_4_ were dissolved together with PPG4000 in acetonitrile/water (43:57, v/v) in the same ratios as in the porogen solutions, followed by light scattering analysis in a Zetasizer Nano ZS (Malvern Instruments, Malvern, UK). A solution containing PPG4000 only in the mixed solvent system was also prepared. This solution appeared turbid and the entities causing this turbidity had a hydrodynamic size larger than the upper measurement limit (10 μm) of the Zetasizer instrument. As a polymer with very limited solubility in water,^32^ PPG4000 apparently underwent hydrophobic aggregation to produce a turbid mixture, even when 43% acetonitrile had been added to the aqueous phase.

Although the acetonitrile/water solutions of F127-OH and F127-PO_4_ both appeared nearly clear, dynamic light scattering (DLS) measurements (Figure S6) revealed that the triblock polyethers self-aggregated due to their amphiphilic nature, to form micelles with hydrophobic PPO cores surrounded by hydrophilic PEO shells.^33^ Interestingly, three peaks were observed in the F127-OH solution, the largest peak corresponding to an average micelle size of 23 nm, but there was also a low intensity peak centered at 790 nm and a signal close to the upper measurement limit at 4.9 μm. DLS studies of poloxamers in aqueous solutions have shown similar patterns, where the peaks corresponding to regular triblock surfactant micelles are accompanied by the peaks of larger clusters of micelles or diblock polymers.^34^ Others have reported that the admixture of ethanol to aqueous solutions of Pluronic F127 does not result in larger micelles, which could explain the cluster signals.^35^ Diblock impurities alleged to be present in commercial poloxamers will also lead to lower critical micelle concentrations and larger and variable aggregation numbers.^36^ The hydrodynamic diameters of the most abundant micelles in the 10 to 50 nm range (median 23 nm) including entrained solvents are well in agreement with aggregation numbers from 37 to 72 in both modeling^37^ and experimental data.^38^ The formation of regular micelles in mixed acetonitrile–water solutions with the organic admixture this high was surprising but is in line with the recent findings by Del Secco et al.^39^ Phosphorylated F127-PO_4_, synthesized for the first time in this work, showed three signals; a smaller peak at 3 nm from an unmicellized block copolymer, a main peak centered at 15 nm (micelles), and also a small cluster peak at 720 nm. The micelles with a median size at 15 nm (ranging from 6 to 30 nm) had the highest relative abundance, suggesting a smaller aggregation number for the F127-PO_4_ with charged terminals, compared to the neutral F127-OH.

When the F127-OH or F127-PO_4_ mesoporogens were combined with the PPG4000 co-porogen in a weight ratio of 9:1, the regular micelles in the 6–50 nm range disappeared entirely for F127-OH and decreased substantially and shifted toward larger diameters for F127-PO_4_, and the small 3 nm peak ascribed to unmicellized F127-PO_4_ vanished. Instead, large and narrow peaks appeared for clusters of F127-OH and F127-PO_4_ with PPG4000, having hydrodynamic diameters of 723 and 767 nm, respectively. This can be explained by the low water solubility of PPG4000, use of acetonitrile as the diluent, and the amphiphilic properties of F127-OH and F127-PO_4_ due to their EO-PO-EO triblock structure. The hydrophobic central PO blocks, accompanied by PPG4000, tend to associate with acetonitrile, with the hydrophilic outer EO blocks oriented toward the more aqueous external phase. Among common aqueous solvent systems, acetonitrile–water has quite unique high deviation from ideal mixing,^40^ with mixtures exhibiting a microheterogeneity manifest in preferential solvation that extends over many solvation shells at room temperature.^41^ The mole fraction of acetonitrile, *x*_S_, in these experiments was 0.234, close to the range where the extremes in the volume-corrected preferential solvation parameters, δ, occur; at *x*_S_ = 0.30 for water–solvent interactions with a negative δ′_WS(min)_ value of −0.37 and at *x*_S_ = 0.35 for water–water interaction with a positive δ′_WW(max)_ of 0.56.^40^ Acetonitrile also has a positive value of +155 cm^3^·mol^–1^ for *G*_SS_^∞^, its self-association at infinite dilution in water, which is the characteristic of relatively hydrophobic solutes that are poor electron pair donors and still miscible with water.^42^ These factors combined the result in a propensity of acetonitrile to shy away from interactions with water and to disperse easily in hydrophobic regions—properties that also contribute to its quality as an eluent in chromatography. The refractive index of pure PPG4000 at 20 °C is 1.451^43^ and that of acetonitrile/water (43:57, v/v) is 1.347.^44^ This explains the haze seen in the solutions with significant amounts of clusters with diameters in the range of the wavelengths of visible light.

**Figure 2:**
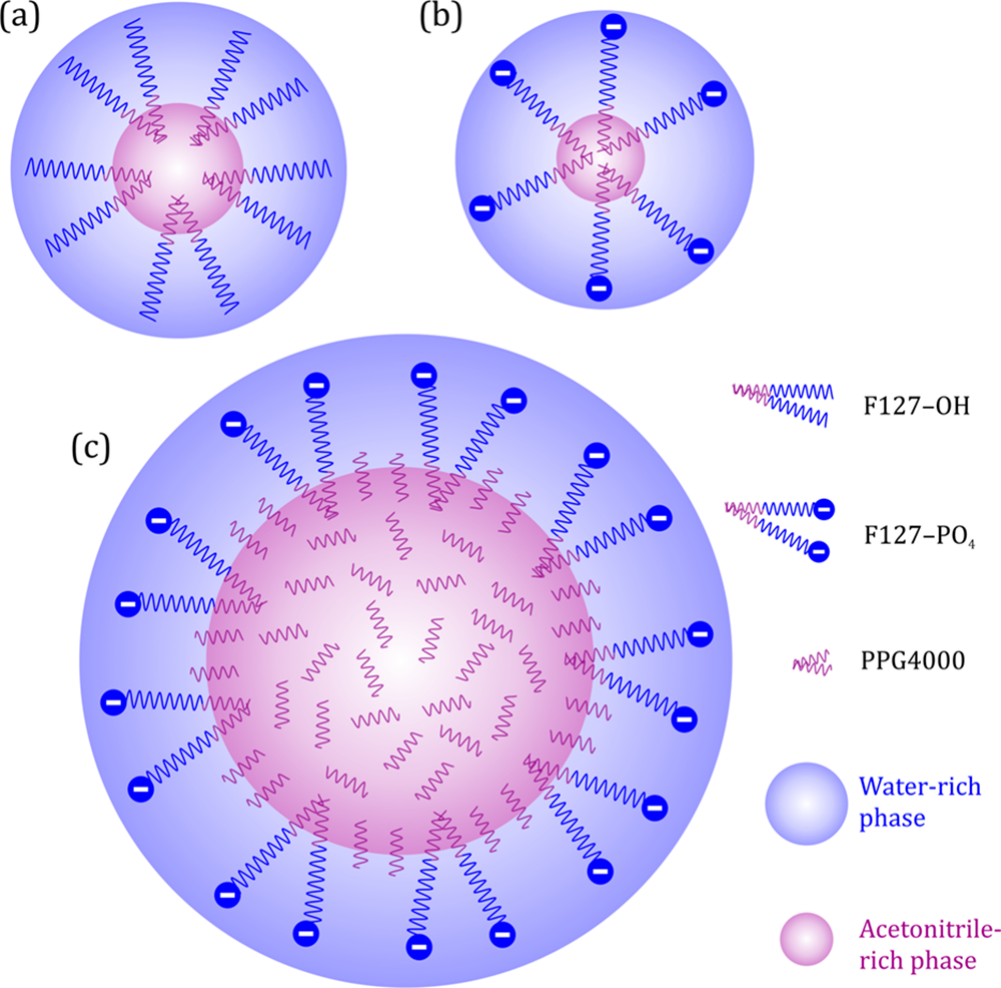
Schematic representations of the proposed micelle structures of the porogen(s) in the acetonitrile/water mixture. (a) F127-OH, (b) F127-PO_4_, (c) mixture of PPG4000 with F127-PO_4_. Blue wavy lines illustrate PEO segments, purple indicate PPO segments of Pluronics.

Moreover, the ζ-potential of the F127-OH/PPG4000 micelles in acetonitrile/water (43:57, v/v) was nearly zero (0.268 mV), whereas that of the F127-PO_4_/PPG4000 micelles was −12.2 mV. The ζ-potentials reported for melamine–formaldehyde microspheres in the literature vary but are generally positive in the absence of electrolytes; Li et al.^45^ reported +45 mV in water, but the same authors^46^ later reported ζ-potentials decreasing from +15 mV to 0 V over the pH range from 6 to 10, which implies that some unspecified electrolyte must have been added. Shen et al.^47^ measured ζ-potentials of MF microspheres with stoichiometry 1:1 (too low for cross-linked network formation) to be +18.7 mV, whereas spheres with stoichiometry 1:7 (formaldehyde in large excess) had a ζ-potential of +36.6 mV. When determined in the medium used in our binding capacity test (1:1 ACN–water, with 0.1% FA), the ζ-potentials of crushed NIP and MIP monoliths were 31.7 ± 2.3 and 36.7 ± 3.3 mV (mean ± SD; *n* = 6), respectively. Both, hence, had strong positive surface charges, with the ζ-potential of the MIP significantly higher than that of the NIP, according to a parametric *t* test (*p* = 0.0074), as well as a nonparametric Mann–Whitney test (*p* = 0.015). Against this background, we can now sketch Figure 2.

Both F127-OH and F127-PO_4_ appear to form regular micelles [(a) and (b)] with their hydrated EO blocks and polar heads extended toward the partly aqueous bulk phase and the central PO blocks tangled up in the micelle cores, shielded from the polar solvent environment. Unlike a purely aqueous system, the acetonitrile diluent will also be preferentially distributed into these micelles, at least to some extent, causing their cores to swell. It is therefore impossible to estimate the aggregation numbers from their hydrodynamic radii. Moreover, each head group occupies a certain space on the micelle surface, and the highly hydrophilic phosphate head groups of the F127-PO_4_ micelles maximize their internal distances by Coulombic repulsion. It is therefore reasonable to assume that each phosphate terminus occupies a larger volume than a hydroxy terminus. Furthermore, for ionic surfactants, Coulomb repulsion tends to counteract the formation of micelles at low concentrations,^48^ which can explain the presence of unmicellized F127-PO_4_ and the smaller hydrodynamic diameter of the micelles formed by F127-PO_4_, compared to F127-OH.

This micellar biphasic porogen system, which reached hydrodynamic diameters in the micrometer range when assisted by the co-porogen PPG4000 (identical to the middle PO block of Pluronic F127) and acetonitrile as the diluent, should have the potential of widening the macropores of the resulting polymer and establish a bicontinuous system when used as the template/porogen with the highly polar aqueous MF precondensate. In this complex, phase-segregated environment, we expected that the opposite surfaces charges of the PPG/F127-PO_4_ micelles and the MF polymer under formation (cf. zeta potential measurements above) would promote an orientation of the highly polar and negatively charged phosphate terminals toward the mainly aqueous precondensate phase, where gradual formation and cross-linking of the positively charged MF polymer takes place. This strengthens the idea of using a terminally modified triblock polyether to simultaneously act as a pore-directing agent and as a template for preparing surface imprinted monoliths with universal selectivity for phosphorylation.

### Modification of the Capillary Inner Surface

A critical factor in the in situ synthesis of efficient and robust monolithic LC columns is to ascertain firm anchoring to the column wall, to prevent uneven density close to the wall, and in extreme cases, even partial detachment, which can cause poor column performance.^49^ We started off with alkaline etching to roughen the capillary wall slightly, followed by acid treatment to convert silanolate ions to free silanols.^28^ From there, we tested three different covalent modifications to ensure well attached monoliths.

The mechanism of melamine polycondensation with formaldehyde in aqueous media at varying pH has been known for a long time.^50^ Briefly, each of the three amine moieties of melamine can react with up to two carbonyl groups to form methylol melamines carrying up to six hydroxymethyl groups,^51^ which are subsequently cross-linked by forming methylene or ether bridges at higher temperatures.^52^ Since monolithic materials tend to shrink on polymerization,^53^ the functional group density on the capillary inner wall has to provide sufficient surface bonding to balance the forces caused by polymeric shrinkage. In this context, an amine–formaldehyde chemistry should be well suited to ensure that the MF prepolymer will wet the fused silica capillary wall and that the monoliths formed upon curing will be strongly linked to it. We therefore investigated three attachment strategies based, as a first step, on covalently bonding amino groups to the fused silica surface.

In the first approach, capillaries **C1** were prepared by silanization with APTES, a standard procedure for the establishment of aminopropyl ligands on silica surfaces. This resulted in well-attached nonimprinted monoliths, with a thin layer of the MF polymer formed on the silica surface to support monolith adhesion (Figure S7a). However, severe detachment was seen for the corresponding MIP, polymerized in the presence of the F127-PO_4_ porogen/template instead of F127-OH (Figure S7b). Since the only difference between these monolith preparations was the phosphate terminals instead of hydroxy terminals on the F127, it is likely that the acidic phosphate terminals protonated the amino groups on the capillary surface and also stabilized the ammonium groups and shielded then from the reaction by ion pair formation, thus rendering the amino groups tethered to the capillary surface unable to participate in the monolith polymerization.

The second and third schemes were developed based on the idea that the reaction with formaldehyde or MF prepolymer could prevent this presumed deactivation and allow the amino group bonded onto the silica wall to be incorporated into the monolithic structure.^52^ In the first of these schemes, the amino groups were methylolated with aqueous formaldehyde (10%, v/v) to produce capillaries **C2**. In a separate experiment, the MF precondensate was diluted 1 + 9 with water, loaded into the aminopropylsilylated capillary, and cured to produce capillaries **C3**. The SEM images of MIP monoliths prepared in **C2** (Figure S7c) and **C3** (Figure S7d) verify that the adhesion of the monolithic frameworks onto the capillary surfaces was adequate both for the NIPs and the MIPs. However, the monolithic structures on the inner wall surface of **C3** appeared more homogeneous than those in **C2**. The reason could be a higher probability of polymer linkage from the capillary surface due to the larger and more flexible structure of methylol melamine prepolymer in **C3** than the azanediyl dimethanol groups of **C2**. Another explanation could be stronger interactions between the activated surface of **C3** and the monolith precursor solution due to hydrogen bonding and/or π–π stacking of the melamine rings. We hence chose the diluted MF precondensate activation to capillaries **C3** in the continued experiments.

### Field Emission Scanning Electron Micrographs

The superficial morphologies of the NIP and the MIP, synthesized in situ in 100 μm ID fused silica capillary **C3**, were probed by FE-SEM, with images shown in Figure S8. Their frameworks appeared largely similar, consisting of fused micron-size spherical particles forming a 3D monolithic structure with some surface roughness, evident at the largest magnification. Convective transport pores are visible between particles, which allowed sustained mobile phase flow at high velocity. No change in backpressure was seen when MeOH was pumped through the monolithic columns at 20 μL/min for 24 h, verifying stable structures.

However, the size distributions of the melamine-formaldehyde particles making up the NIP and MIP monoliths were dissimilar. Histograms of the diameters of 200 fused particles each from the NIP and MIP, measured on SEM images by ImageJ,^54^ showed that the NIP monolith appeared to follow a bimodal distribution (a Shapiro–Wilk normality test^55^ on the 200 data points gave *p* = 0.02219) centered on 1.72 and 2.54 μm, while the diameters of the particles forming the MIP material were normally distributed (Shapiro–Wilk *p* = 0.4747) with median at 2.27 μm (Figure S9a,b). The only composition difference in the cocktails used to prepare the NIP and the MIP was the conversion of the hydroxyl end groups of Pluronic F127 to anionic phosphate groups to act as the imprinting template for the MIP. However, end-group modifications are known to have a substantial impact on the polymer morphology.^56^ Moreover, the negative charge of the phosphorylated terminals will engage in electrostatic interactions with protonated amino groups during the gel-forming stage of the polymer formation, and the reduced surface tension caused by the biphasic monomer/porogen mixture is expected to promote the formation of imprints, assisted by additional matching geometries of complementary polar groups as the viscosity of the system increases with the degree of polymerization.

Both the NIP and MIP monolith surfaces appeared to be textured, according to the FE-SEM images. This, combined with the broad distributions in mesopore sizes characterized by nitrogen cryosorption, ranging from 2 to 50 nm with maxima around 30 nm for the NIP and 20 nm for the MIP (Figure S9c,d), confirm the mesopore-forming role of F127-OH and F127-PO_4_. Specific surface areas of 13.3 ± 4.0 and 7.3 ± 1.9 m^2^/g for the NIP and MIP (*n* = 3), respectively, show that the mesopore content in the monolithic structures was as expected, considering the relatively large mesopore sizes, which will be advantageous to provide good mass transfer of larger molecules, such as polypeptides.

### Fourier-Transform Infrared Spectroscopy

Nonimprinted and imprinted polymers prepared from excess monolith cocktail in vials together with the capillary monoliths were characterized by FT-IR (Figure S10). Peaks at 3349 and 754 cm^–1^ are associated with N–H stretching and deformation vibrations of secondary amine and peaks at 1548 and 814 cm^–1^ belong to the triazine ring stretching. The 1357 cm^–1^ peak is attributed to the C_aromatic_–N stretching vibration and the broad peak centered at 3423 cm^–1^ is due to the stretching vibrations of O–H. Methylene C–H stretching, bending, and twisting vibrations are evident at 2958, 1492, and 889 cm^–1^, respectively. Stretching vibrations of C–O–C are seen at 1016 and 1159 cm^–1^. The practically identical FT-IR spectra of the NIP and MIP, along with a total absence of a P=O stretching signal at 1114 cm^–1^ in the MIP spectrum, showed that both the materials had matching chemical compositions and that the F127-PO_4_ template/porogen had been completely removed.

### X-ray Photoelectron Spectroscopy

Surface elemental stoichiometries of the NIP and MIP were determined by XPS with survey spectra shown in Figure S11. The corresponding elemental compositions are listed in Table S2, along with binding energies and atomic abundances for the analyzed peaks. Again, the nearly identical survey spectra illustrated the similarity in C, N, and O elemental composition between the NIP and MIP materials, with strong signals from C 1s (≈288 eV) and N 1s (≈400 eV), and a signal of lower intensity at ≈533 eV corresponding to O 1s. The atomic percentages of C and N were 46.61 and 49.99 for the NIP and 47.10 and 48.66 for the MIP, which translates into corresponding C:N ratios of 0.93:1 and 0.97:1, respectively. These C:N ratios are reasonable and agree well with the molecular structure of MF polymers (Figure 1b), where the melamine ring has six nitrogens and three carbons, and the melamine–formaldehyde molar ratio in the precondensate used to prepare the monoliths was 1:2.93, leading to an expected C:N ratio of (3 + 2.93):6 = 0.98:1.

Referring from here on to the expanded XPS spectra in Figure S12, the C 1s component resolved at 285.0 eV is assigned to [**C**–(C, H)], the 286.8 eV peak to [**C**–(O, N)], the peak at 287.8–287.9 eV to the combined signals from [N=**C**–N] and [**C**=O], and 293.8–293.9 eV to C 1s [π–π* excitation]. Finding signals from [**C**–(C, H)] was not expected since all the carbons in an ideal MF resin (Figure 1b) should be bonded to nitrogen or oxygen (or both). Still the spectra reveal significant [**C**–(C, H)] signals of 1.66 atom % in the NIP and 2.96 in the MIP. This could be suspected to emanate from the methyl groups of the PPG 4000 or the PO segment of Pluronic F127-OH or F127-PO_4_ if they had been incompletely washed out after the synthesis. However, the C:N ratios found by XPS were lower than expected for both the NIP and the MIP, so this scenario can therefore not explain the **C**–(C, H) signals. Another explanation for this could be surface contamination, which is very difficult to avoid even when meticulous care is taken in the sample preparation step. If the C:N ratios are recalculated without the [**C**–(C, H)] carbon, the C:N ratios for the NIP and the MIP will be 0.90:1 and 0.91:1, respectively, which show the close similarity in chemical composition between NIP and MIP. The lower-than-expected C:N ratio can then be due to the known loss of formaldehyde from MF resins during the conversion of ether to methylene linkages.^57^

Considering the N 1s signals for the NIP and the MIP, the two N 1s peaks appearing at 398.6–398.8 eV and at 399.9–400.0 eV are attributed to [C=**N**–C] of the triazine rings and [**N**–(C, H)] for imino bridges or terminal amino groups, respectively. Their ratio should also ideally be 1:1 since no nitrogen except melamine is added to the polymerization mixture. The results 1.06:1 for the NIP and 1.00:1 for the MIP (Table S2) are in good agreement with expected numbers. The explanation for these somewhat deviating ratios could again be sought in contamination, or simply the relatively high measurement uncertainty inherent in XPS measurements on surfaces of high fractal dimension, which is related to high BET surface areas.^58^ Moreover, the absence of detectable signals from P 2p around 133 eV in the survey spectra of the imprinted MIP monoliths (Figure S11) further verifies that phosphorylated triblock polyether acting as the template and surfactant had not been linked to the MIP and that the washing procedure after imprinting had successfully removed the F127-PO_4_ template from the surface layer to a level that was undetectable by XPS.

In terms of total oxygen atomic percentages, there are two contributing peaks assigned to O 1s, at 530.5 eV for [C=**O**] and 532.8–532.9 eV for [(C–**O**H), (C–**O**–C)]. The [C=**O**] signals were absent in the NIP and low as expected in the MIP since carbonyl groups are not expected to be formed in the polycondensation reaction. It is finally possible to evaluate the ratio of residual ether bridges and unreacted methylol groups [(C–**O**H), (C–**O**–C)] to [**N**–(C, H)] from the melamine amino groups. The results from the NIP and MIP were similar, 0.14:1 and 0.18:1, respectively, showing that the MF monoliths are well cured since most methylol groups seem to have been reacted and the ether linkages initially formed in the cross-linking of melamine have mainly been further reacted to methylene bridges.

### Nuclear Magnetic Resonance

CP-MAS ^13^C NMR spectra of the NIP and MIP monoliths also verified their highly similar chemical composition (Figure S13) and prove that the porogen/templates (F127-OH and F127-PO_4_) involved in the imprinting and pore formation did not take part in the polymerization. The intense peaks at 166.35 ppm from triazine ring carbons^59^ verify that both polymers are composed of melamine rings covalently cross-linked via methylene bridges (−N–**C**H_2_–N–; two peaks at 48.78 and 54.63 ppm), and ether bridges and/or methylol groups (−N–**C**H_2_–O–**C**H_2_–N– and/or–N–**C**H_2_–OH, two signals at 65.51 and 72.34 ppm).

To sum up, the FT-IR, XPS, and CP-MAS ^13^C NMR data confirm that the melamine had reacted with paraformaldehyde and was cross-linked during the thermal curing, resulting mainly in the formation of methylene bridges with lower amounts of methylene ether bridges and residual methylol groups, in agreement with previous curing studies of melamine–formaldehyde resin.^52,60^ No spectroscopic signals attributable to porogens or template were observed and all spectral features of the NIP and the MIP were remarkably similar. The equivalent chemical compositions of the NIP and the MIP, and the efficient removal of porogens and templates, have consequently been confirmed. We therefore conclude that the selectivity differences between the NIP and MIP accounted for in the following experiments and chromatographic evaluations must be due to spatial surface conformations of the monolith surfaces, established by the different templates used for the NIP and the MIP, and not due to differences in their chemical composition.

### Batch Binding Isotherm Assays

The affinities and capacities of the monolithic materials were investigated by a binding isotherm method applied to ground bulk adsorbents, with phenylphosphoric acid (PPA) and Fmoc-**pY**-OEt as test probes in 50% (v/v) aqueous acetonitrile. This acetonitrile admixture is in the same range as the present polymerization solvent and within the range used in common high-performance liquid chromatography (HPLC) mobile phases, which means that the imprinting efficiency will be tested under realistic eluent conditions. The fitted Langmuir mono-site adsorption models (lines in Figure S14) for the bound/free isotherm curves of the PPA test probe both had correlation coefficients >0.99 and described the adsorption of PPA well, with notable differences in binding properties between the NIP and the MIP monolithic materials. The adsorption capacity (*B*_max_) values in Table S3 derived from these models and specific surface areas based on N_2_ cryosorption measurements indicate accessible active binding site densities of 57.1 ± 2.0 and 203 ± 11 μmol/m^2^ for the NIP and the MIP, which results in an imprinting factor (eq S6) of 3.56. These absorption capacities are, however, far too high to be explained by monolayers, and this excess surface coverage is likely a result of electrostatic interactions due to the opposite charges of the MF material and the probes, as elaborated above. The uptake of Fmoc-**pY**-OEt was not as excessive, although the *B*_max_ values of 11.4 ± 1.6 and 25.1 ± 4.1 for the NIP and MIP are both higher than that could be expected from monolayer coverage, yielding an imprinting factor or 2.20. The fitting of Langmuir isotherms to the Fmoc-**pY**-OEt data was worse than that to PPA (*R*^2^ = 0.9323 and 0.8902 for the NIP and the MIP, respectively). Parts of the reason for this are the three points with the lowest free Fmoc-pY-OEt concentrations in the MIP curve, which show a remarkably steep initial rise, hinting at a substantially higher selectivity at concentration levels more realistic for use as trapping columns in LC–MS.

Polymeric monolithic materials based on cross-linked melamine could engage in a variety of interactions with analytes carrying phosphoric acid moieties. Electrostatic interactions will be active between singly dissociated phosphate groups and protonated secondary amine residues under the acidic test condition (pH ≈ 3.0; 0.1% formic acid in 50% ACN^61^) of the batch binding assays. Hydrogen bonding and dipolar interactions should also be in effect, thanks to the high ratio of nitrogen in the polymeric backbone. The plenitude of sites capable of engaging in Coulombic and polar interactions also led to nonspecific interactions, evident from the adsorption capacity also of the NIP toward PPA. However, the conversion of the hydroxyl terminals of the amphiphilic block copolymer in the prepolymerization cocktail of the MIP into phosphoric acid groups seems to have created an abundance of complementary cavities with higher affinity and selectivity toward phosphorylated analytes in the polymerization step.

### Chromatographic Evaluation

The selectivity of the MIP in the trapping of phosphorylated peptides was first investigated by tracing the retention patterns of the peptide GADDSYYTAR, nonphosphorylated, and mono-phosphorylated on either of the tyrosines with a tryptic digest of BSA added to challenge the MIP with an excess of peptides without phosphorylation.

**Figure 3:**
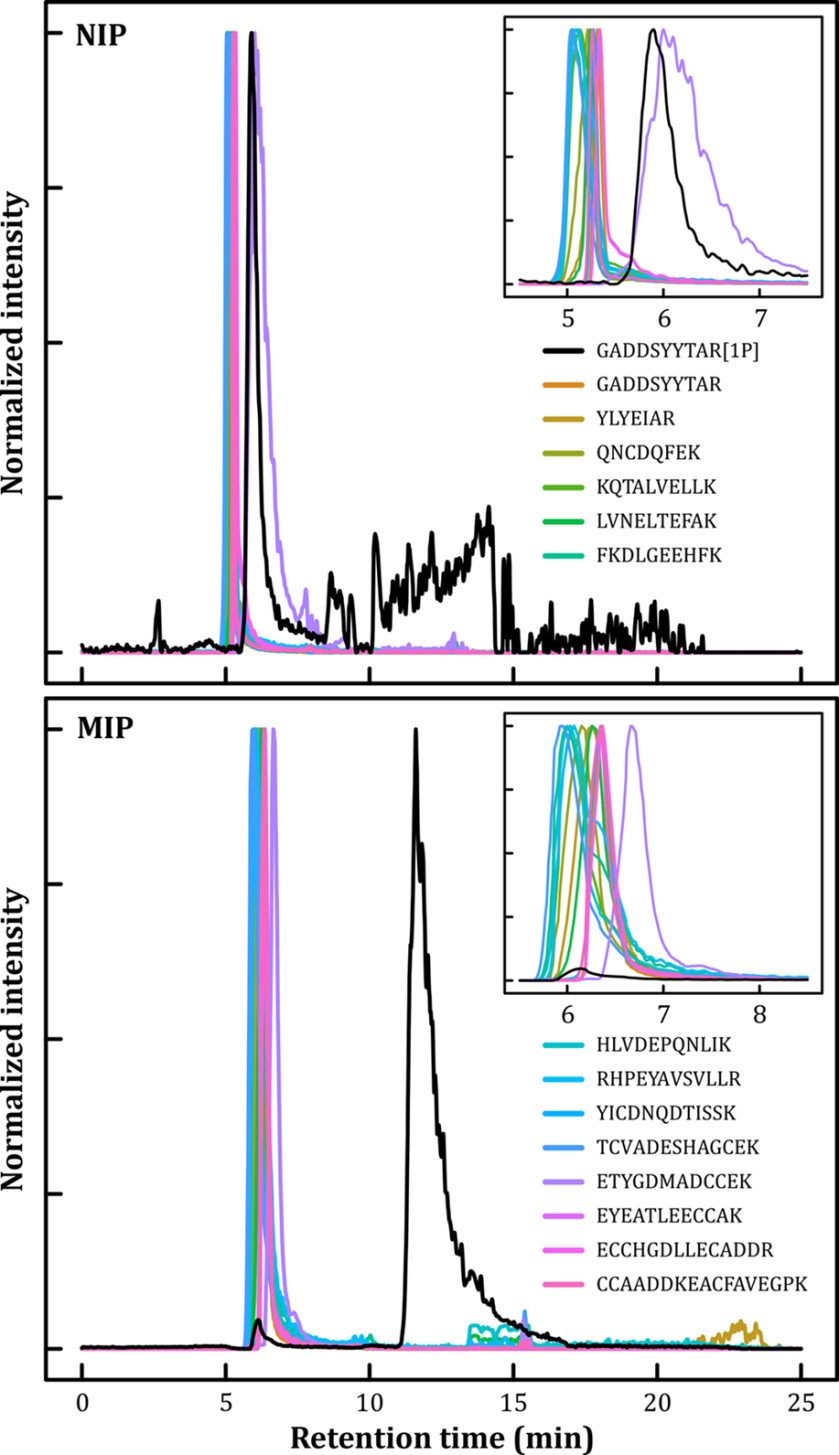
Extracted ion chromatograms of spiked ZAP70 peptides, GADDSYYTAR, and GADDSYYTAR (1P, mono-phosphorylated) in tryptic digest of BSA, including 15 peptides detected by MASCOT with SwissProt database, separated on NIP and MIP monolithic capillary columns. Extracted chromatograms were filtered by the Savitzky–Golay algorithm (*n* = 1, *p* = 7) to reduce the heteroscedastic shot noise caused by the operation of the electrospray under high acetonitrile conditions, and thereafter normalized to unit max intensity. Peptide legends have been split between the plots and are valid for both. Colored version of the plot is available in the on-line version.

It is obvious from the extracted ion chromatograms in Figure 3a that all the identified nonphosphorylated BSA peptides co-eluted independent of their molecular weight in a retention window from 5.0 to 6.1 min, as narrow peaks of a symmetric or slightly tailing shape, when injected on the NIP column and eluted by a multi-step gradient of increasing ACN percentage (Figure S15). The 70 mm long 0.25 mm i.d. capillaries have a total volume of 3.4 μL, and according to the monolith recipe, the maximum mass fraction of the MF polymer in the column was 0.21. With an MF polymer density of 1.56,^62^ the void time should be around 2.9 min at the mobile phase flow rate of 1 μL/min. When extra-column void volumes are accounted for, it can therefore be concluded that the tested peptides co-eluted on the NIP column quite close to the void volume, meaning that there was a lack of specific recognition toward nonphosphorylated peptides when tested against a tryptic digest containing peptides with a wide span in composition and size. It should be noted that a large fraction of the mono-phosphorylated GADDSYYTAR peptides also eluted as a sharp peak with slightly longer (≈0.7 min) retention time compared to the bulk of the nonphosphorylated tryptic peptides from BSA, although some excess retention was seen on the NIP (the normalized extracted ion signal is noisy since the sample eluted over a large fraction of the gradient). Both these observations are in good agreement with the bound/free isotherm assays, where the NIP material offered some binding capacity toward phosphorylated probes. Although the imprinting effect (IF = 2.20) may not be as high as compared to other imprinted materials,^2,3^ the use of molecularly imprinted monoliths showed their strengths for liquid chromatographic separation, which enables selective targeting of the analytes of interest.

As mentioned above, the cross-linked melamine structure offers a variety of nonspecific interactions for side chains and terminals of peptides, including electrostatic, dipolar, or hydrogen bonding. This led to the longest retention time on the NIP column for ETYGDMADCCEK (see inset in Figure 3a), the BSA peptide among those identified with the highest abundance of acidic side chain amino acids; two each of Asp and Glu, and only a single basic side chain Lys, yielding a theoretical pI of 3.92. Some weak anion exchange therefore seems to contribute to the mixed mode interaction mechanism of peptides on the nonimprinted melamine-based materials in the ACN/water medium.

When the same peptide mixture was injected onto the MIP column (Figure 3b), the nonphosphorylated peptides, including ETYGDMADCCEK, showed retention patterns similar to those on the NIP, with retention times ranging from 5.9 to 6.7 min. In contrast, the probe GADDSYYTAR monophosphorylated on tyrosine eluted as a sharp peak centered at ≈12 min with a short albeit characteristic “MIP tailing”.^63^ Hardly any signal was seen near the void volume where the nonphosphorylated peptides eluted, proving a specific recognition toward the phosphate group.^64^ A peak width of around four minutes indicates reasonably homogeneous binding sites and decent mass transfer rates, which we attribute to surface imprinting by the F127-PO_4_ template. Despite this unconventional approach of simply tethering phosphate groups to the terminals of an amphiphilic block copolymer to act as “epitopes” for phosphorylated amino acids side chains, it appears to have created an MIP with a highly selective binding of phosphorylated side chains over carboxylic groups, even when the phosphorylated side chains are located well away from the peptide terminals, as in the GADDSYYTAR peptide.

### Modeling of Phosphorylated Peptide Recognition by Projection to Latent Structures

We continued to probe the affinity and selectivity of the MIP by a more realistic sample, composed of the combined tryptic digests of twelve common proteins, using an LTQ Orbitrap XL MS instrument. These tryptic peptides enabled us to obtain MIP and NIP retention patterns for a range of phosphorylated and nonphosphorylated peptides of varying lengths. Seventy of these peptides (Table S4) were chosen based on having peak intensities >10^5^ CPS after Savitzky–Golay filtering of their extracted ion chromatograms to reject spurious signal spikes. When identified by the ExPaSy database, the peptides were from non- to tri-phosphorylated, covering a mass range from 1000 to 3800 Da. The retention times of these peptides were used as the explained (*Y*) variable in a projection to latent structures (PLS) model in Umetrics SIMCA 16.0 (Sartorius, Umeå, Sweden). The explanatory (*X*) variables first tested were the molecular weight, number of phosphorylations, and the counts of each amino acid in the peptide sequence as scaled numerical variables. This led to a single component model with decent correlation (*R*^2^ = 0.357), but without prediction capability, evident from its low cross-validation score (*Q*^2^_max_ = 0.047). A new PLS model was therefore established with a reduced number of explanatory variables. The molecular weight and phosphorylations were kept, but instead of using the number of individual amino acids in each peptide, they were bundled into the total counts of basic (R, H, and K), acidic (D and E), hydrophilic (S, T, N, and Q), and iodoacetamidated (C) side chains and used as scaled numerical variables in a new PLS model. Aliphatic index^65^ was also added to represent amino acids with hydrophobic sidechains (A, V, I, and L). The first fitting attempt indicated a single outlier, peptide 70-3P, which was eliminated from the set of observations.

**Figure 4:**
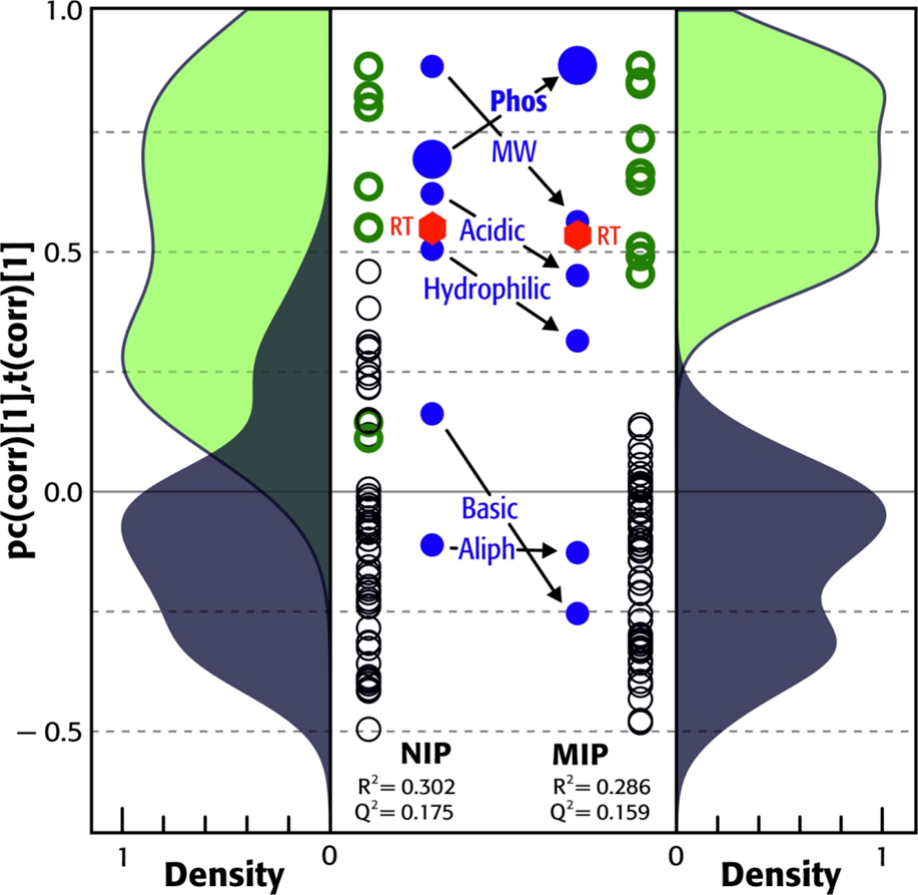
Comparison of biplots of scores and loadings of the projection against latent structures (PLS) models for NIP and MIP capillary columns of the retention of nonphosphorylated and phosphorylated peptides (black and green open circles, respectively) found in a tryptic digest of twelve proteins, using as explaining variables aliphatic index (Aliph), the molecular weight (MW), the number of phosphorylations (Phos), basic, acidic, and hydrophilic side chains, all plotted as filled blue circles. Red hexagons are retention time (RT), the modeled variable. Flanking the plotted variables are normalized Gaussian kernel density plots of the identified nonphosphorylated and phosphorylated peptides, in dark gray and light green, respectively.

A biplot of loadings and scores for the NIP and the MIP resulting from the new single component PLS model, which gave maximum predictive power according to cross-validation, is shown in Figure 4. This visualizes the connections between some fundamental peptide parameters and the observed retentions of the identified peptides in a single plot. The PLS models of both the NIP and MIP columns revealed decent correlations with peptide retention times of 30 and 29%, respectively, expressed as the *R*^2^ of the models at maximum predictability, *Q*^2^.

For the NIP column, the four factors contributing most strongly towards explaining the retention time were molecular weight (89%), phosphorylation (70%), and the bundles of acidic (62%) and hydrophilic (51%) side chains. The basic side chains had a weak positive (16%) contribution to the retention time, which is somewhat surprising considering the positive surface potential of the MF monolith (see above), whereas the aliphatic index had a weak negative correlation (−11%). The cross-linked melamine base with a carbon to nitrogen atomic ratio of 1:1 (confirmed by XPS) should be capable of entering into several types of polar and electrostatic interactions with peptide side chains. The PLS model corroborates this mixed-mode mechanism of peptide binding on the NIP column, and the slight excess retention of multi-carboxylic side chain peptide ETYGDMADCCEK discussed above is explained by the positive correlation of acidic side chains with the retention time. Phosphorylation was also one of the main factors, rationalized by the positive surface charge of the MF monoliths and the acidic test conditions (0.1% FA in ACN/water),^61^ which should promote electrostatic binding. Molecular weight was the strongest retention-promoting factor, which can be at least partly explained by the relation between their residue counts and molecular weights. A linear regression of molecular weight against the number of acidic, basic, and hydrophilic groups showed an *R*^2^ of 0.64, with slope highly significant at the 99.9% level.

A positive correlation between the molecular weight and retention also rules out significant contributions from size exclusion effects. Peptides in the current 1 to 4 kDa MW range are macromolecules with unfolded hydrodynamic radii from ≈ 0.8 to 1.7 nm.^66^ Size exclusion effects could therefore occur for materials with high fractions of micropores.^67^ The cross-linked melamine monoliths presented here have the pore size distributions in the mesopore range (2–50 nm, peaking at 20–30 nm) and the connected macropore system seems to have allowed good access for all tested peptides.

When the same tryptic peptide mix was separated on the MIP column, the correlation between the peptide retention and phosphorylation increased from 70 to 89%, while the correlations with all other descriptors except the aliphatic index decreased sharply; molecular weight from 89 to 57%, acidic side chains from 62 to 45%, hydrophilic side chains from 51 to 32%, and basic side chains from 16 to −26%. This verifies that recognition sites selectively capable of trapping of phosphopeptides had been formed by the terminally phosphorylated block copolymer template. The success of the imprinting step was also evident from the promotion of retention by the MIP material only for phosphorylation, but not for carboxylic acid side chains. The unaffected lack of contribution of the aliphatic index indicates that hydrophobic interactions were not part of the retention mechanism on the MF materials.

Finally, to evaluate if other descriptor variables were suppressed by the strong contribution of phosphorylation on the MIP, we fitted another PLS model with all samples included, excluding phosphorylation as an explanatory factor. This model had *R*^2^ and *Q*^2^ values of 0.066 and −0.012 (although the value is termed *Q*^2^, it can attain negative values due to the way it is calculated in SIMCA), respectively, and could not be validated, confirming that apart from phosphorylation, none of other variables had any systematic explanatory effect on the peptide retention on the MIP column.

Based on the full model of peptide retention on the MIP column, a hierarchical cluster model classified well all the identified peptides into two major clusters; nonphosphorylated and phosphorylated (Figure S16). On a lower dendrogram level, the distances between observations were arbitrary, which emphasizes the role of phosphorylation on peptide binding toward the MIP material. For the NIP column, the first cluster level consisted of peptides with phosphorylation or molecular weight above 2 kDa. The second cluster consisted of the remaining nonphosphorylated peptides with molecular weight below 2 kDa. At the lower level, the combination of molecular weight and acidic and hydrophilic groups played key roles in the cluster formation.

It should be pointed out that the number of amino acids with carboxylic groups did not affect the peptide binding when formic acid was used as a mobile phase additive, which is an advantage over phosphopeptide enrichment by metal-based materials.^4^ Hydroxyl and amide side chains also lacked influence on the retention times, although hydrogen bonding is expected to play an important role in imprint rebinding. Another advantage of using cross-linked melamine is the lack of retention bias from hydrophobic parts of the peptides; see Aliphatic Index in Figure 4. Although melamine is classified as an aromatic amine, its electrons are more located on the nitrogens than on the carbons, leading to electrostatic interactions being more favorable than π–π interactions.^68^

## Conclusions

A terminally phosphorylated triblock polyether was synthesized via a facile single-step route and used to produce a molecularly imprinted monolith based on hydrophilic melamine–formaldehyde in the capillary format. By performing the dual roles of the selectivity-promoting template and the pore-forming agent, this phosphorylated poloxamer established a group selectivity for phosphate groups. The affinity and selectivity have been tested and modeled by reliable methods, such as thermodynamic bound/free isotherm, chromatographic evaluation, and projection to latent structures. The monolithic MIP capillary columns have been evaluated with real tryptic digested proteins, under conventional mobile phase gradients of increasing ACN percentage in mixture with water and formic acid as an additive. This MIP material is therefore a potential recognition material for phosphorylated peptides with possible applications in various areas of the biological science complex, namely, phospho-proteomic analysis, clinical diagnosis, and so on.

## Supporting Information Available

Additional reagents; background information; and descriptions and results from the characterization experiments in Figures S1–S16 and Tables S1–S4 (PDF)
