# Supplementary material for: Terminally Phosphorylated Triblock Polyethers Acting Both as Templates and Pore-Forming Agents for Surface Molecular Imprinting of Monoliths Targeting Phosphopeptides
Source: ACS Omega. 2023 Feb 20;8(9):8791–803. doi: 10.1021/acsomega.3c00007 (PMC9996590; doi:10.1021/acsomega.3c00007)
Supplement: Supplementary file 1 — ao3c00007_si_001.pdf [file ao3c00007_si_001.pdf]

Supporting Information

Terminally Phosphorylated Triblock Polyethers Acting both as Templates and Pore-forming Agents for Surface Molecular Imprinting of Monoliths targeting Phosphopeptides

Chau Minh Huynh[a*], Ignacio Arribas Díez[b], Hien Kim Le Thi[a], Ole N. Jensen[b], Börje Sellergren[c], Knut Irgum[a]

a) Umeå University, Department of Chemistry, S-901 87 Umeå, Sweden.
b) University of Southern Denmark, Department of Biochemistry & Molecular Biology and VILLUM Center for Bioanalytical Sciences, Campusvej 55, DK-5230 Odense M, Denmark.
c) Malmö University, Faculty of Health and Society, Department of Biomedical Science, S-205 06 Malmö, Sweden.

Table of Contents

Characterization and Evaluation Procedures 3
Preparation of Trypsin Digested Proteins 3
Nitrogen Cryosorption 3
Field-Emission Scanning Electron Microscopy 3
Fourier-transform Infrared Spectroscopy 3
X-ray Photoelectron Spectroscopy (XPS) 3
HPLC-MS/MS method 3
Solid-state Nuclear Magnetic Resonance Spectroscopy 3
Determination of Polymer Composition using Nuclear Magnetic Resonance Spectroscopy 3
DLS and Zeta Potential Measurement 4
Binding Isotherm Assay 4
Capillary Liquid Chromatography 4
Multivariate Data Analysis 5
Figure S1. Scheme showing the reactions used to modify the capillary inner surfaces 5
Figure S2. ^{31}P NMR spectra of (a) L61-PO_4, (b) L121-PO_4, (c) P123-PO_4, and (d) F127-PO_4 6
Figure S3. FT-IR spectra of Pluronic F127–OH and its phosphorylated derivative, F127–PO_4 6
Figure S4. ^{1}H, and ^{13}C NMR spectra of Pluronic F127-OH and phosphorylated Pluronic F127-PO_4 7
Figure S5. ^{31}P NMR spectra of F127–PO_4 7
Figure S6. Hydrodynamic diameter distribution of mesoporogen and co-porogen/mesoporogen mixture 7
Figure S7. FE-SEM images of monoliths in capillaries with different inner surface modifications 8
Figure S8. FE-SEM images of NIP and MIP capillary monoliths at different magnifications 9
Figure S9. Pore characterization and histograms of fused particle diameters in the NIP and MIP monoliths 10
Figure S10. FT-IR spectra of NIP and MIP monoliths 11
Figure S11. Survey XPS spectra of NIP and MIP monolith surfaces 11
Figure S12. High-resolution C1s, N1s, and O1s, XPS spectra of NIP and MIP monoliths 11
Figure S13. CP-MAS ^{13}C-NMR spectra of NIP and MIP monoliths 12
Figure S14. Binding curves for PPA and Fmoc pY OEt after incubation with NIP and MIP. 12
Figure S15. Step-gradient solvent program used in the in LC-MS evaluations 13
Figure S16. Hierarchical cluster analysis of 69 tryptic peptides identified using the NIP and MIP capillaries 13
Table S1. Properties of the Pluronics included in the initial template synthesis efforts 14

* Corresponding author. Phone: +46 76 0866539; e-mail: chau.huynh@umu.se

Table S2. Surface elemental composition of MF monoliths by XPS 14
Table S3. Langmuir monosite fitting of NIP and MIP with PPA and Fmoc pY OEt as probes 14
Table S4. List of peptides tryptic detected identified by LC-MS 15
References 16

Characterization and Evaluation Procedures

Preparation of trypsin digested proteins. Twelve proteins containing phosphorylated residues were used to prepare tryptic digest samples: Carbonic anhydrase (bovine), serum albumin (bovine), ribonuclease B (bovine pancreas), lactoglobulin (bovine), α-casein (bovine), β-casein (bovine), ovalbumin (chicken), lysozyme (chicken), alcohol dehydrogenase (*Saccharomyces cerevisiae*), myoglobin (whale skeletal muscle), α-amylase (*Bacillus* sp.) and transferrin (human). All proteins were from Sigma-Aldrich except transferrin, which was from ACE Biosciences (Odense, Denmark). Each protein was separately dissolved in 50 mM triethylammonium bicarbonate buffer to a concentration of 20 pmol/µL, reduced with 10 mM of 1,4-dithiothreitol at 56 °C for 30 min, and subsequently alkylated with 40 mM of iodoacetamide for 30 min at room temperature, protected from light. Trypsin (1 % of the protein mass) was thereafter added followed by incubation at 37 °C for 12 h. The protein digest sample was prepared by mixing peptides from each of the protein digests in equimolar ratio and diluting with 0.1 % aqueous trifluoroacetic acid to final concentrations of 2 pmol/µL for each protein. This peptide mixture was stored at –20 °C until further use.

Nitrogen Cryosorption. Extracted and dried monolith cubes with ≈ 2 mm sides (50 to 150 mg) recovered from the vials were transferred to dry 9.5 mm i.d. sample tubes and further dried for 2 h at 60 °C by a flow of dry N_2 using a Micromeritics SmartPrep degassing unit (Atlanta, GA, USA). The samples were then immediately subjected to multipoint nitrogen adsorption-desorption analysis at cryoscopic temperature on a Micromeritics TriStar 3000 automated gas adsorption analyzer. The specific surface areas of the monolithic materials were determined based on the Brunauer–Emmett–Teller (BET) model[S1] using adsorption data in the relative pressure range from 0.18 to 0.35. The Barrett–Joyner–Halenda (BJH) scheme[S2] was used to estimate the total pore volume, average mesopore diameters between 1.7 and 300 nm, and pore size distribution based on the desorption branches of the isotherms, shown in Figures S9c-d.

Field-Emission Scanning Electron Microscopy. Samples with fresh breakage rupture surfaces were positioned on adhesive carbon foils attached to standard aluminum sample stubs and mechanically secured to the holders by conductive glue (Ted Pella Inc., Redding, CA, USA). All samples were thereafter coated with a 10 nm of layer of platinum in a Quorum Q150TS sputter coater (Quorum Technologies, Ringmer, UK). The metal-coated samples were analyzed in a Zeiss Merlin field emission scanning electron microscope from Carl Zeiss Microscopy GmbH (Oberkochen, Germany) operated at 5 kV acceleration voltage. Images were captured from randomly chosen areas at standardized magnifications.

Fourier-transform Infrared Spectroscopy. Diffuse reflectance Fourier transform infrared spectroscopy (DRIFTS) was performed on a Bruker (Ettlingen, Germany) IFS 66 FTIR spectrometer under ≈ 400 Pa partial vacuum. Dried monoliths (≈ 10 mg) were ground manually together with ≈ 390 mg of KBr using an agate mortar and pestle followed by direct transfer to a DRA-2CI diffuse reflectance cell from Harrick Scientific Products (Pleasantville, NY, USA). The signals were recorded using a total of 256 co-added interferogram scans, transformed to obtain spectra between 4000 and 400 cm^{-1} at a spectral resolution of 4 cm^{-1} with acceptable signal-to-noise ratio.

X-ray Photoelectron Spectroscopy (XPS). Surface elemental analysis and elemental stoichiometries were determined on dried monoliths by means of XPS. A Kratos Axis Ultra DLD electron spectrometer (Kratos Analytical, Manchester, UK) equipped with a monochromatic Al $K_{\alpha 1}$ X-ray source (1486.6 eV) operated at 150 W and a delay-line detector was used in the measurements. The surface potential was stabilized by the charge neutralization system of the spectrometer. All binding energies (BE) are referenced to the C 1s C-H peaks of aliphatic carbon at 285.0 eV and at 284.7 eV for aromatic carbon. Processing of the spectra was accomplished with the Kratos software.

HPLC-MS/MS Method. Reversed phase HPLC was used to determine the unbound amounts of test compounds in the isotherm experiments. The column was an ACE-III C_{18} (150 × 2.1 mm, 5 µm) from Advanced Chromatography Technologies (Aberdeen, UK) and separations were done in isocratic mode. When the target compound was PPA, the mobile phase consisted of 0.1 % formic acid in acetonitrile/water 1:1 (v/v), pumped at a flow rate of 200 µL/min. Mass spectrometric detection was performed on an LCQ Duo ion-trap mass spectrometer (Thermo Finnigan, San Jose, CA, USA), in negative ion mode with quantitative fragment set to m/z = 173.00 and qualitative fragment set to m/z = 79.00. For Fmoc-**pY**-OEt, the mobile phase consisted of 35 vol-% acetonitrile in 10 mM aqueous ammonium formate. Mass spectrometric detection was performed in positive mode, with quantitative fragment set at m/z = 501.10 and qualitative fragment at m/z = 483.70.

Solid-state Nuclear Magnetic Resonance Spectroscopy. Extracted, dried, and ground monolith powder was transferred to 4 mm inner diameter zirconium oxide (ZrO_2) NMR rotors with Kel-F inserts. Cross-polarization magic angle spinning ^{13}C NMR (^{13}C CP-MAS NMR) was performed on a Bruker (Billerica, MA) Avance III 500 MHz spectrometer, equipped with a ^{13}C CP-MAS probe, spun at a rate of 10.0 kHz. Spectra were acquired at 298 K with a 2.85 µs proton 90° pulse, followed by a cross polarization step using a ^{13}C spin lock field strength of 62.5 kHz and a ramped ^{1}H field strength (from 43 to 86 kHz) during 1.5 ms. ^{1}H decoupling was done by the SPINAL64 sequence[S3] using a decoupling field strength of 88 kHz during the 6.8 ms acquisition time. The FID signals were collected with 2 s relaxation delay and 3000 scans and the accumulated FID signals were multiplied by a Gaussian apodization function prior to Fourier transform, followed by manual phase and baseline correction. Adamantane was used as an external chemical shift reference with CH_2 signals set to 38.5 ppm. All spectra processing was performed using Bruker TopSpin 4.0.6.

Determination of Polymer Composition using Nuclear Magnetic Resonance Spectroscopy. Experiments were carried out at 298 K using a Bruker AVIII 400MHz spectrometer equipped with a 5 mm SmartProbe BBF-H/D. Pluronics F127-OH and F127-PO_4 (0.100 ± 0.001 g) were dissolved in separate 500 µL aliquots of a 80:20 (v/v) mixture of acetonitrile-d3 and deuterium oxide. Spectra were referenced using the acetonitrile proton peak at 1.94 ppm. The **H**DO peaks appeared at 3.84 ppm with F127-OH and at 4.15 ppm with F127-PO_4 (Figures S4a and

c). This can be compared to a shift of 3.66 ppm observed by Koley and Ghosh for $\chi_{H_2O} = 0.42$[S4]. Spectra were recorded by Bruker TopSpin 4.0.6, using 1024 zg30 sequence pulses at 5 s intervals with receiver gain of 90.5, 5 s relaxation delay, 10.4 µs pulse width, and 7978.7 Hz spectral width.

A detailed composition analysis of Pluronics using NMR spectrum is illustrated below.

(i) Normalize methyl proton of PPO at 1.05 ppm to 3.0

(ii) Determine the relative moles of PPO (denoted as x)

$$x = \frac{\text{Methyl proton integral}}{3} = 1 \quad \text{[S1]}$$

(iii) Determine the relative moles of PEO (denoted as y)

$$y = \frac{\text{Methylene and methanetriyl proton integral}}{4 \times y + 3 \times x} \quad \text{[S2]}$$

(iv) Calculate the weight percentages of PEO and PPO

$$\%w_{\text{PEO}} = \frac{44 \times y}{44 \times y + 58 \times x} \times 100 \quad \text{[S3]}$$

DLS and Zeta-potential Measurement. The cluster sizes and zeta potentials of the mixed porogens in a biphasic solvent mimicking polymerization condition were both measured at 60 °C using a Zetasizer Nano ZS (Malvern Instruments). A 633 nm He-Ne laser was used as light source and an avalanche photodiode (APD) as detector. The porogen cluster sizes were measured using the dynamic light scattering method, where the scattered light was collected at 173°. The values are reported as the median cluster diameters, in plots of hydrodynamic diameter (nm) *vs.* intensity (%). Zeta potential measurements were based on the electrophoretic light scattering principle. A disposable folded capillary cell DTS1070 from Malvern Instruments was used and signals were collected at 12.8°. All data were analyzed by Zetasizer Software 7.13. The Smoluchowski model was used to calculate the hydrodynamic diameters and the zeta potentials were calculated by the default method in the Zetasizer software. Measurements were made in the porogen solution prepared as described in the experimental section, to which Milli-Q water was added in ratio of 1:1.3 to mimic the polymerization system. Additionally, three solutions, each only containing one of the porogen mixture components (PPG4000, F127-OH, or F127-PO_4), were prepared in the same ratio to investigate their individual roles in the micelle and cluster formation.

Binding Isotherm Assay. The crushed and sieved monolithic materials (5 mg each of MIP and NIP) were separately mixed in 1.5 mL screw cap vials with 1 mL of phosphorylated probe PPA at varying concentrations in acetonitrile/water 1:1 (v/v) containing 0.1 % (w/w) formic acid, then shaken overnight on a Vibrax VXR Orbital Shaker (IKA-Werke, Staufen, Germany). The samples were then centrifuged for 3 min at 2800 × g relative centrifugal force, and the supernatants were analyzed by the RP–HPLC–MS/MS method described above to determine the concentrations of the unbound probes. The amount of bound analyte per unit surface area of polymer (B) was calculated according to

$$B = \frac{(C_0 - C) \cdot V}{m \cdot S}, \quad \text{[S4]}$$

where C_0 and C are the probe concentrations of the initial solution and supernatant, respectively, V is the total volume of the adsorption mixture, m is the mass, and S the specific surface area of the polymer in each vial.

Binding curves were established by plotting B against C, and fitting these by non-linear regression in OriginPro 2020 (OriginLab Corporation, USA) to a Langmuir mono-site model,

$$B = \frac{B_{max} \cdot K_{eq} \cdot C}{1 + (K_{eq} \cdot C)}, \quad \text{[S5]}$$

where B_{max} is the maximum amount of probe bound to each surface area unit, and K_{eq} is the dissociation constant.

Imprinting factors (IF) were calculated by the ratios of saturated uptake of the MIP and NIP, as

$$IF = \frac{B_{max}(MIP)}{B_{max}(NIP)}. \quad \text{[S6]}$$

Capillary Liquid Chromatography. LC-MS evaluations of the capillary monolithic columns were done by loading test mixtures of non-phosphorylated and phosphorylated peptides onto a 300 µm i.d. × 5 mm C18 PepMap 100 trap column (5 µm, 100 Å, Thermo Scientific, Waltham, MA, USA), followed by transfer of these peptides onto the monolithic capillary acting as separation column. The LC-MS set-up consisted of an Ultimate 3000 HPLC system connected to an LTQ Orbitrap XL mass spectrometer, both from Thermo Scientific. The outlets from the capillary monoliths were connected directly to 20 µm PicoTip emitters (New Objective, Woburn, MA, USA) to produce a nano-electrospray. Mobile phase A (MPA) consisted of 0.1 % (v/v) formic acid (FA) in water, and mobile phase B (MPB) consisted of 95 % acetonitrile, 4.9 % water, and 0.1 % FA. In the loading step, 2 % of MPB was used to capture all peptides to the C18 trap column before eluting them onto the monolithic capillary column by 20 % of MPB for 2 min. The peptides loaded onto the capillary monolith were separated by a gradient with increasing percentage of MPB: 20 % (5 min), 20–35 % (0.5 min), 35 % (5.5 min), 35–60 % (0.5 min), 60 % (5.5 min), 60–95 % (3 min), and 95 % (5 min). The gradient is shown in Figure S15.

Two testing mixtures were designed for investigation of the selective enrichment of phosphorylated peptides onto the MF capillary monoliths. The first contained a mixture of GADDSYYTAR peptides (non- and mono-phosphorylated on tyrosine (YY, and pYY or YpY), at final concentrations of 250 fmol/µL of each) spiked into a tryptic digest of 500 fmol/µL bovine serum albumin (BSA) to act as a realistic matrix. The second test mixture, aimed to provide multivariate data, consisted of a pooled tryptic digest of twelve proteins (2 pmol/µL in total), which included numerous naturally phosphorylated tryptic peptides.

The capillary monoliths were equilibrated with at least 100 µL of 20 % MPB before each injection of 1 µL test sample dissolved in MPA. Separations were performed at a total eluent flow rate of 1 µL/min with the gradient composition given above. Blanks of MPA were injected between each sample to minimize carry-over. Mass spectrometric detection was performed in positive ion mode with data dependent acquisition (DDA, top 4), fragmentation induced by higher-energy collisional dissociation (HCD, 29 eV). The mass tolerance of the precursor ion was set to 5 ppm.

Data were acquired as RAW files by Xcalibur 2.5.5 SP1 (Thermo Scientific), from which extracted ion

chromatograms (EICs) based on exact monoisotopic m/z values for the respective peptides were extracted by the *msaccess* module of ProteoWizard 3.0.20337 (64-bit)[S5]. Carbamidomethyl (C) was selected as fixed and phospho (S, T, and Y) as variable modifications. A Savitzky-Golay filtering (3rd order, 15 points) was implemented by the *sgolayfilt* function from version 0.7-6 of the *signal* package[S6] in the 64-bit version 4.0.2 of R[S7]. EICs having filtered peaks with intensity higher than 10^5 were used for further data analysis.

Multivariate Data Analysis. Modeling of peptide properties against their retention was done by projection against latent structures (PLS) in SIMCA 15.0 (Umetrics, Umeå, Sweden), using aliphatic index[S8], molecular weight, and the total number of residues with phosphorylated, basic, acidic, and hydrophilic side chains as explanatory variables. As explained variable the peptide retention times were chosen, corrected by subtracting the system dwell time determined by substituting an empty capillary for the monolith-filled one. All variables were centered and scaled to unit variance. The peptide sequences, and their given codes (where *e.g.* "2" means the second non-phosphorylated peptide and "58-1P" means the 58th peptide phosphorylated at a single site; see Table S4) as Primary IDs. The PLS models were extracted with single components since further component extractions did not improve the cross-validation score, Q^2. Biplots of loadings and scores were produced using the built-in procedure in SIMCA to visualize the connections between samples, factors, and responses in a single plot. The kernel density estimates for the non-phosphorylated and phosphorylated peptides in Figure 4 were computed separately with the *density* function in R, using a Gaussian kernel at the default bandwidth, followed by normalization to unit magnitude. A hierarchical cluster analysis was finally applied to analyze the separation of natural tryptic peptides on the NIP and MIP monolithic columns.

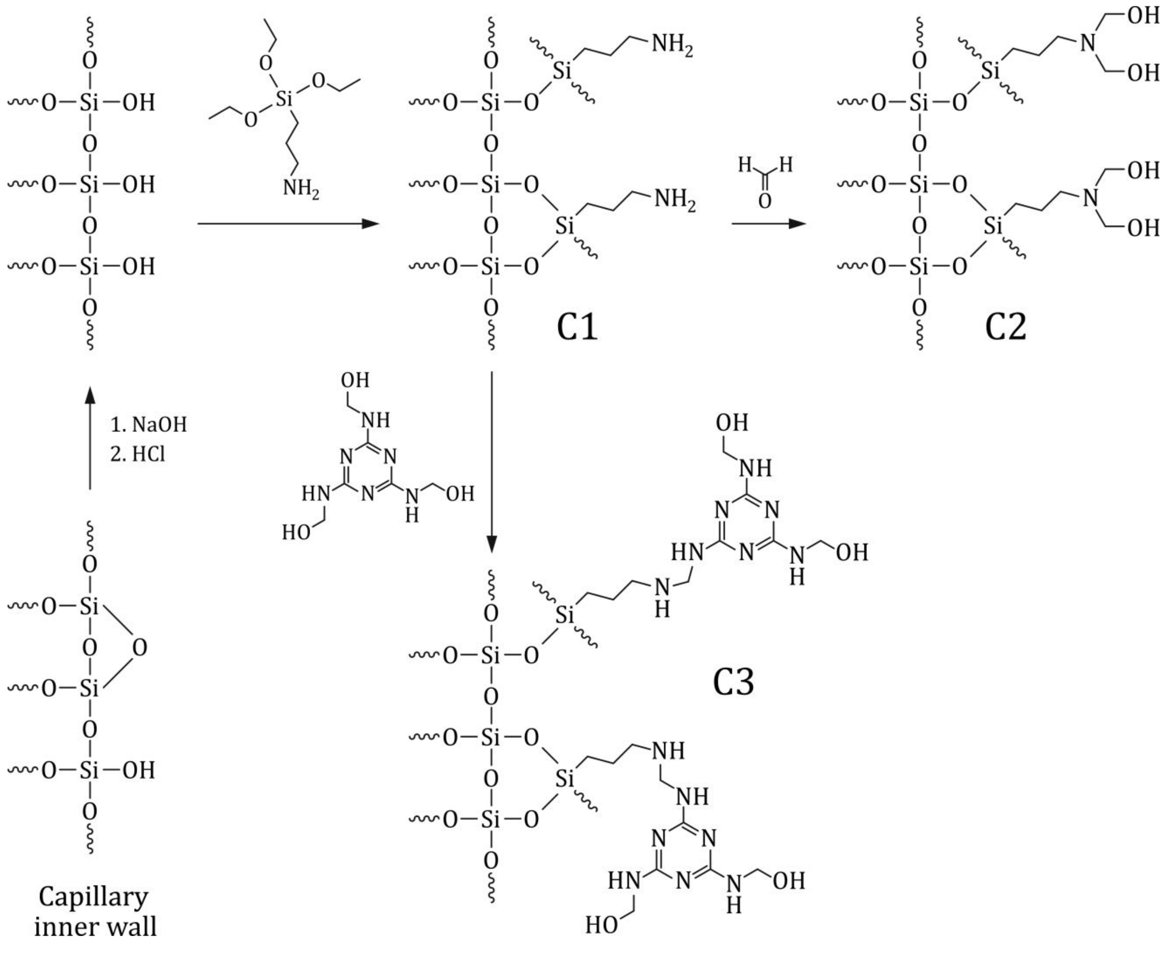

Figure S1. Modifications of the capillary inner surfaces. The fused silica surface is first etched, then the siloxane groups are converted into silanol groups that react with ethoxysilanes groups to form 3-D attachment of amine to the surface (**C1**). Then, **C1** was treated by formaldehyde or diluted trimethylol melamine pre-polymer to produce capillaries **C2** or **C3**, respectively.

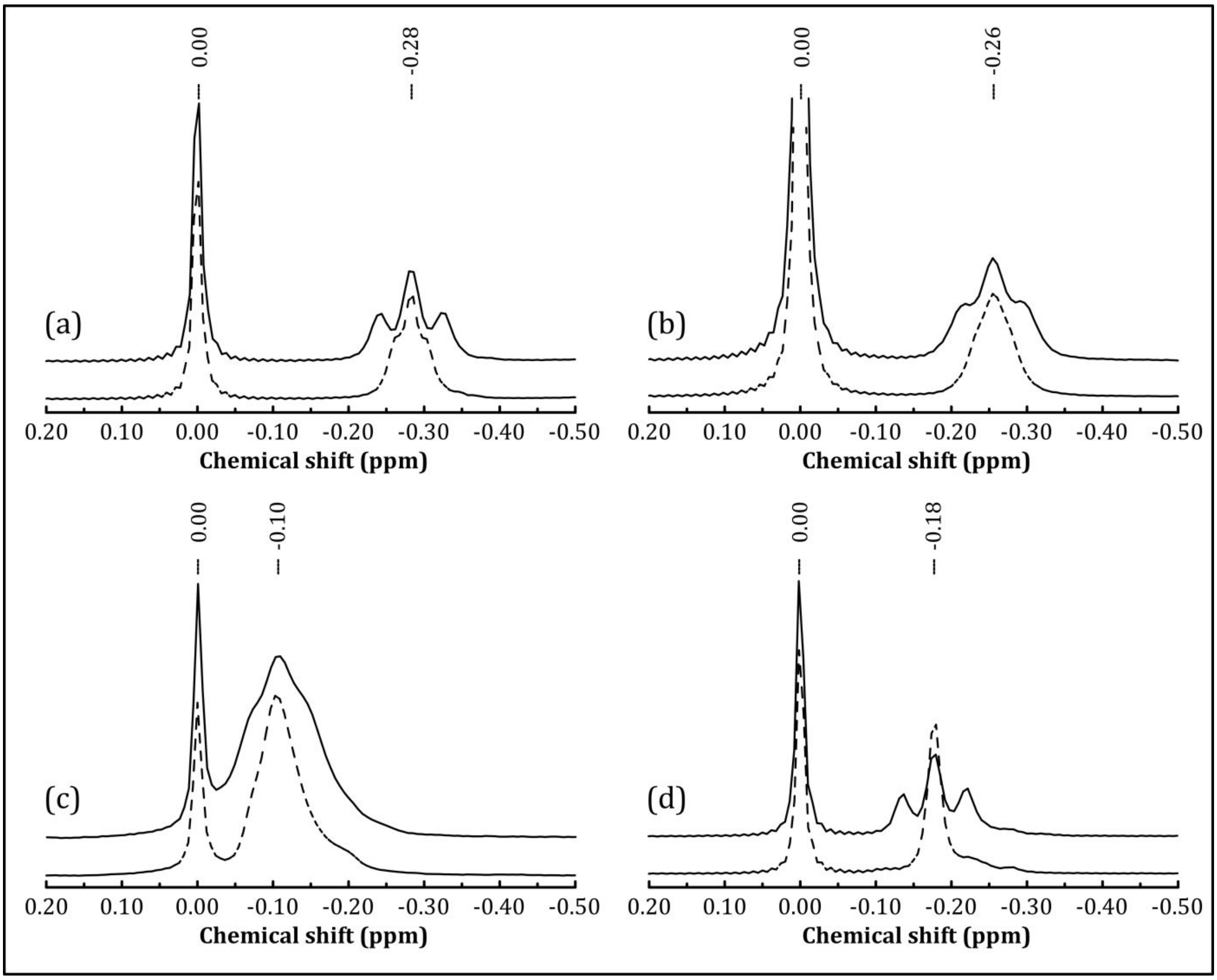

Figure S2. ^{31}P NMR spectra of (a) L61-PO_4, (b) L121-PO_4, (c) P123-PO_4, and (d) F127-PO_4 in coupling (solid line) and decoupling (dashed line) modes with two adjacent hydrogens. The reference peak of H_3PO_4 is at 0 ppm.

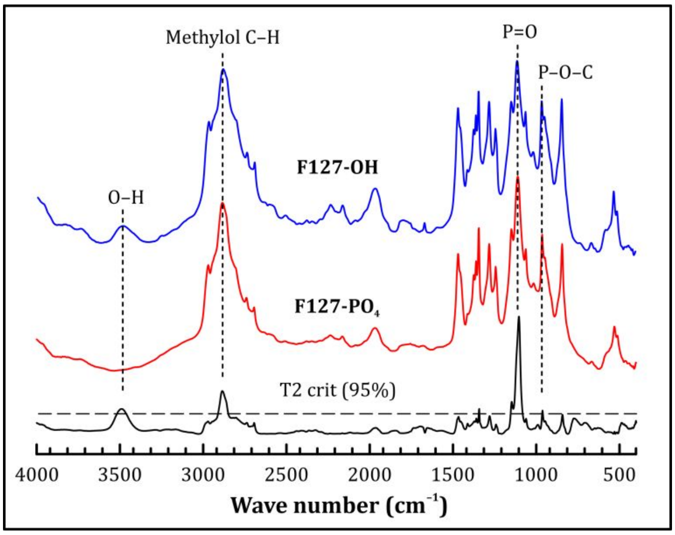

Figure S3. FT-IR spectra of Pluronic F127–OH and its phosphorylated derivative, F127–PO_4. The bottom trace is a Hotelling's T^2 line plot from a single component principal component analysis of the normalized spectra with 95 % confidence interval, indicating where the spectra are significantly different.

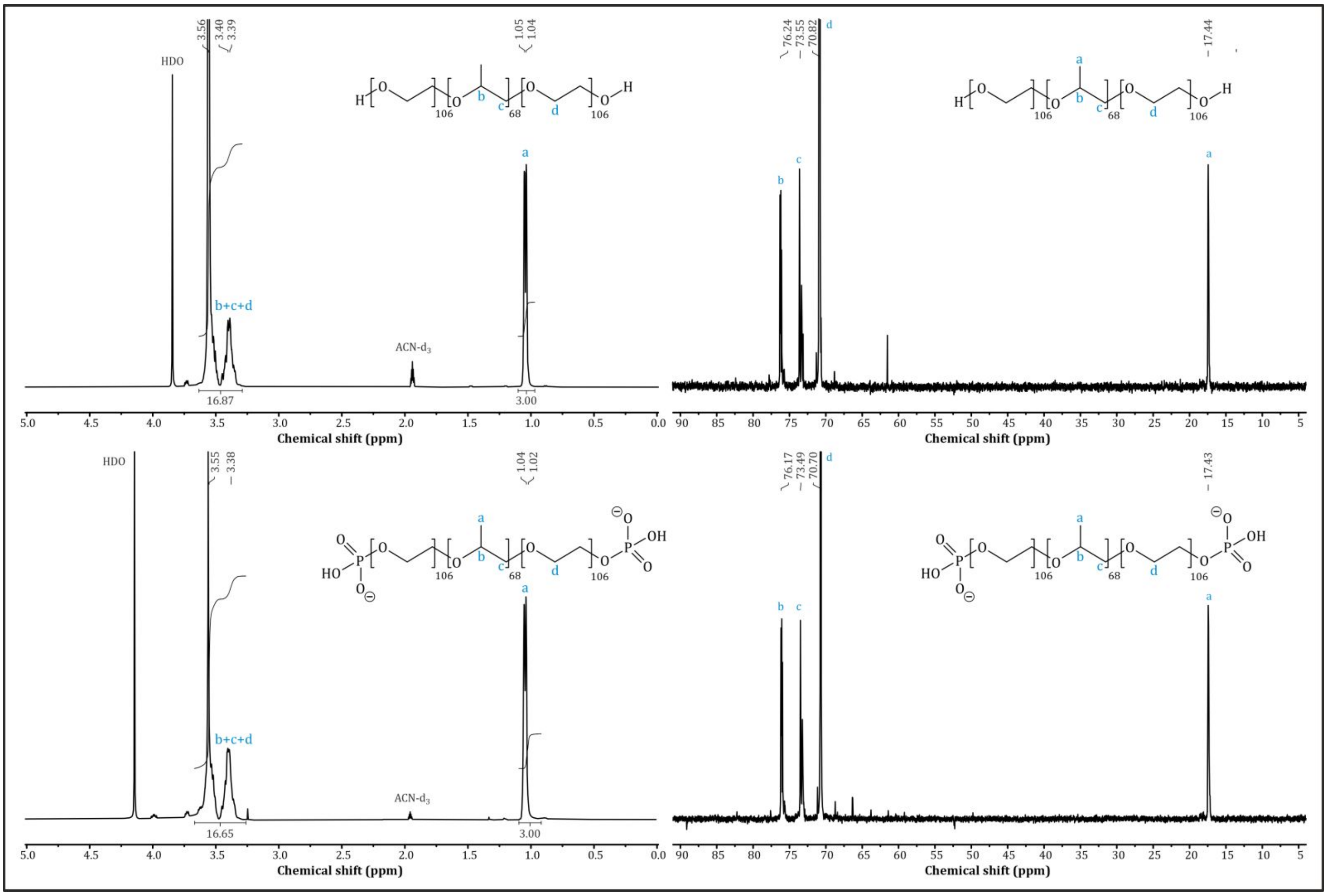

Figure S4. ^{1}H, and ^{13}C NMR spectra of (a), (b) Pluronic F127-OH, and (c), (d) phosphorylated Pluronic F127-PO_4. The solvent mixture used in the measurements was 80 % (v/v) of acetonitrile-d3 in D_2O.

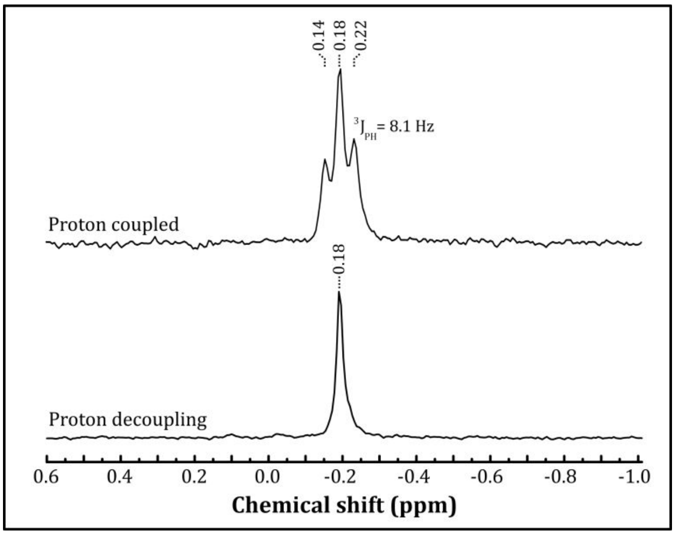

Figure S5. ^{31}P NMR spectra of F127–PO_4, showing a triplet peak in ^{1}H coupling mode, and singlet peak in ^{1}H decoupling mode.

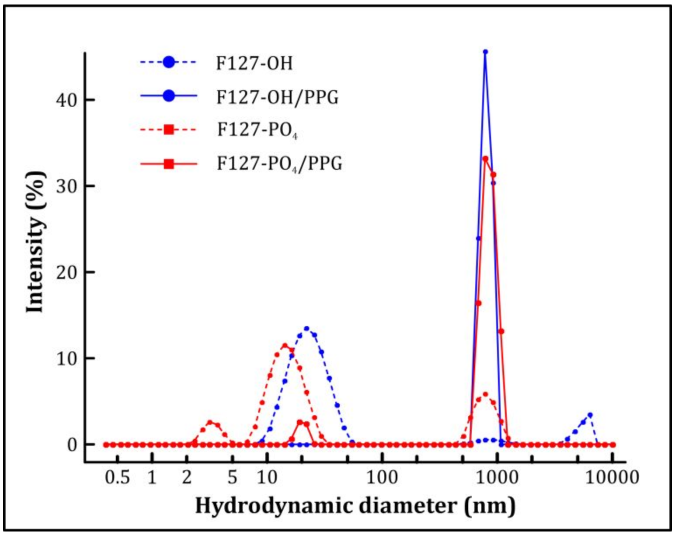

Figure S6. Hydrodynamic diameter distribution of mesoporogen (dashed lines) and co-porogen/mesoporogen mixture (solid lines) in polymerization solvents.

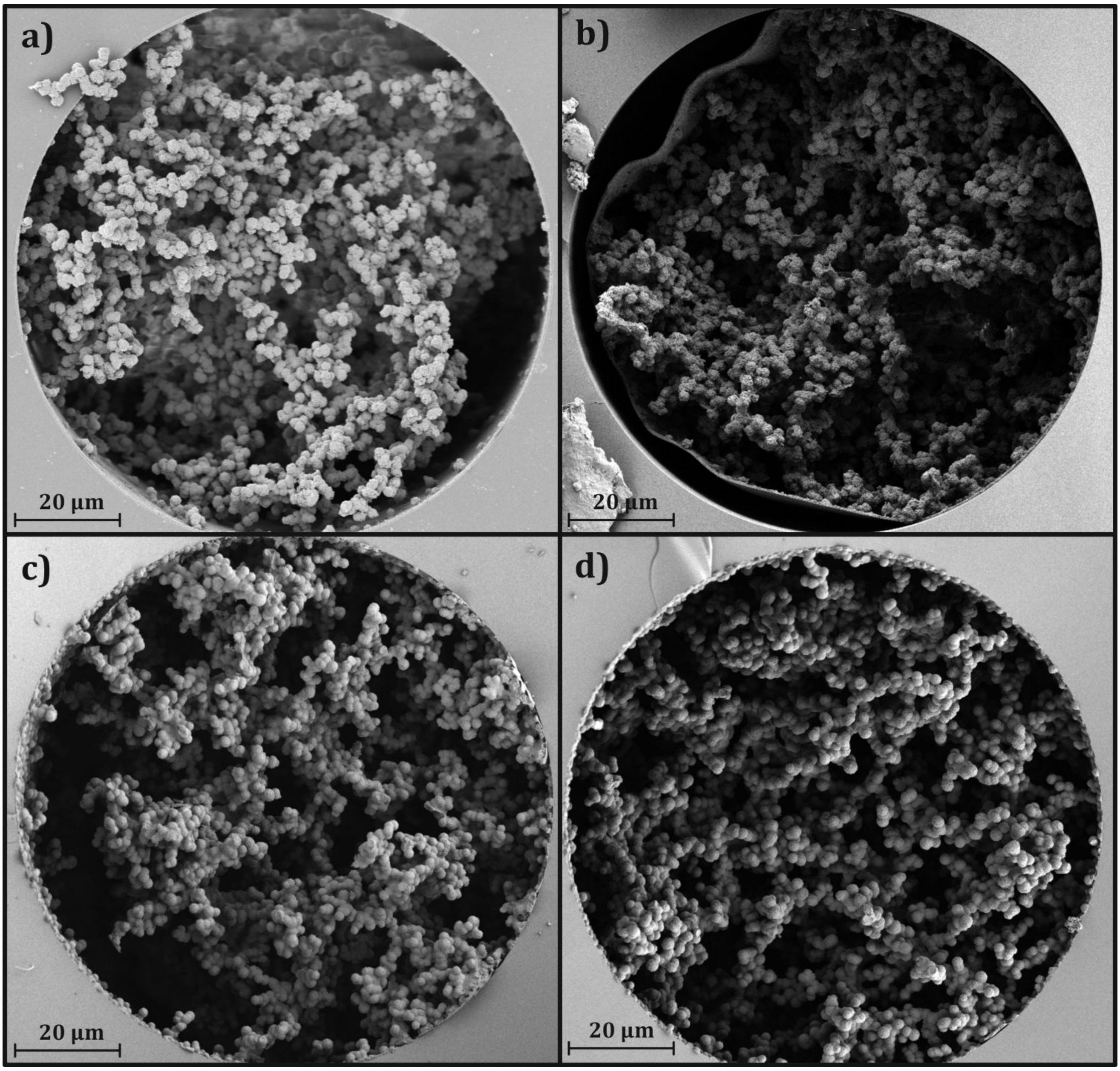

Figure S7. Field emission scanning electron micrographs (FE-SEM) of random fracture cross-sections of monoliths in capillaries with different inner surface modifications: (a) NIP and (b) MIP in capillaries aminated by APTES (capillary **C1**); (c, d) MIP in capillaries aminated by APTES further reacted with aqueous formaldehyde solution (capillary **C2**; c) and diluted MF precursor (capillary **C3**; d). Images have been contrast adjusted to cover the entire greyscale.

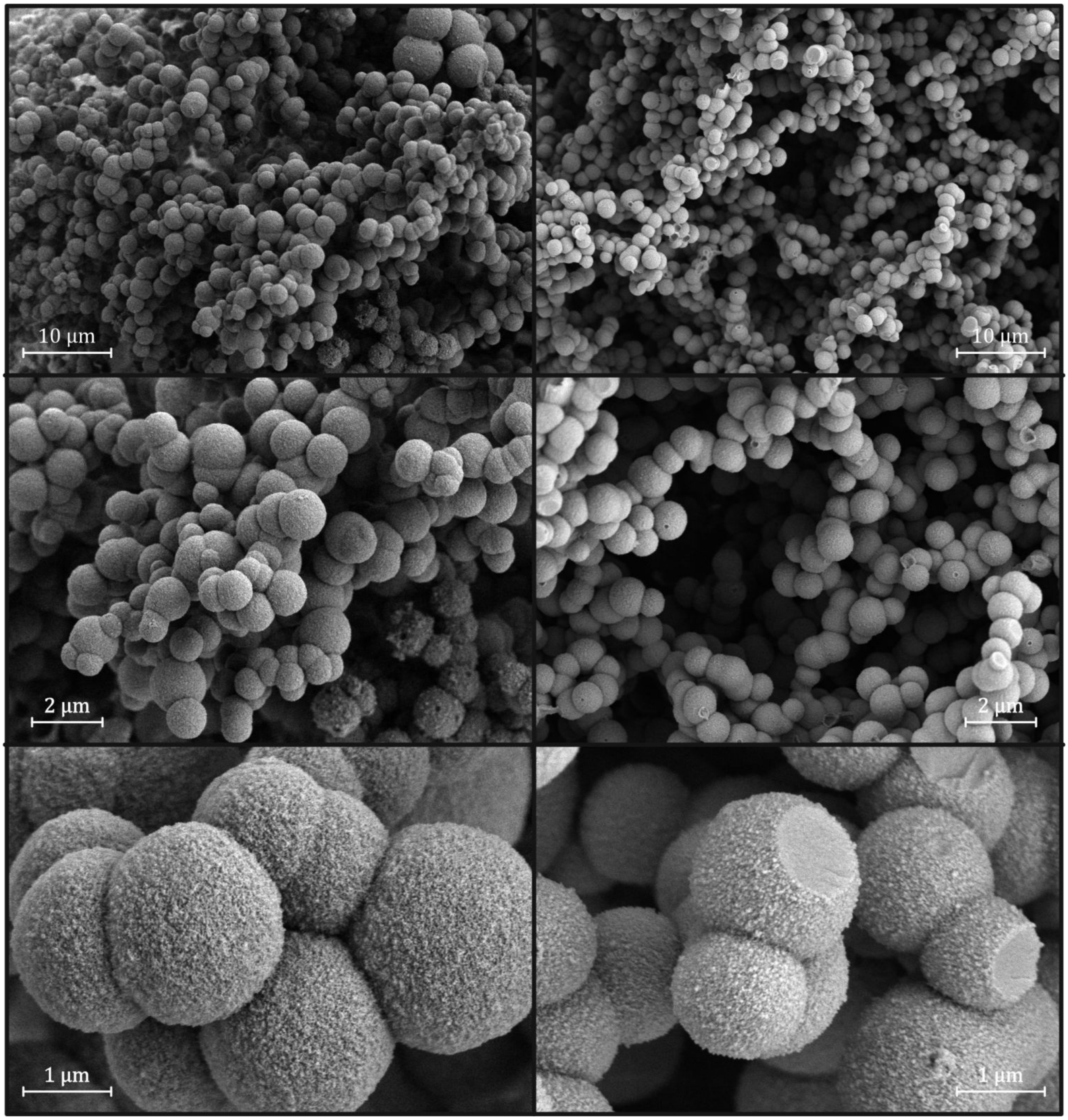

Figure S8. Field emission scanning electron micrographs (FE-SEM) of random fracture cross-section surfaces of (left) NIP, and (right) MIP monoliths in capillaries at 5k, 20k, and 50k times magnifications. Images have been contrast adjusted to cover the entire greyscale.

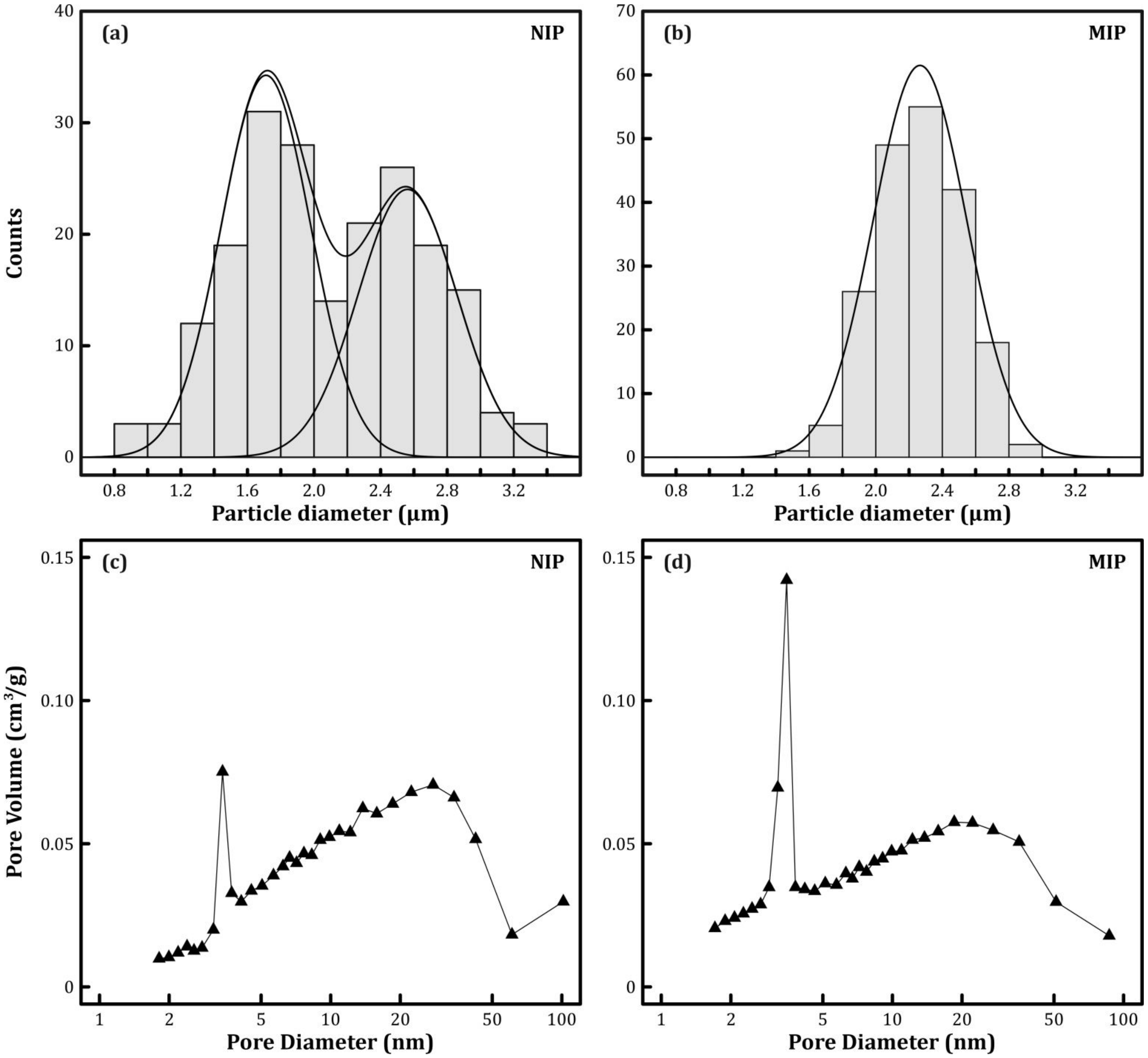

Figure S9. (a-b) Histograms of fused monolithic particle diameters (n = 200) for the NIP and MIP monoliths, respectively. The particle diameters were measured by ImageJ (NIH, USA) and fitting of normal distribution functions to the histograms was done with non-linear least squares (NLS) fitting in R, using the Gauss-Newton algorithm; (c-d) BJH pore volume plots dV/dlog(D) from the desorption branch of nitrogen cryosorption isotherms.

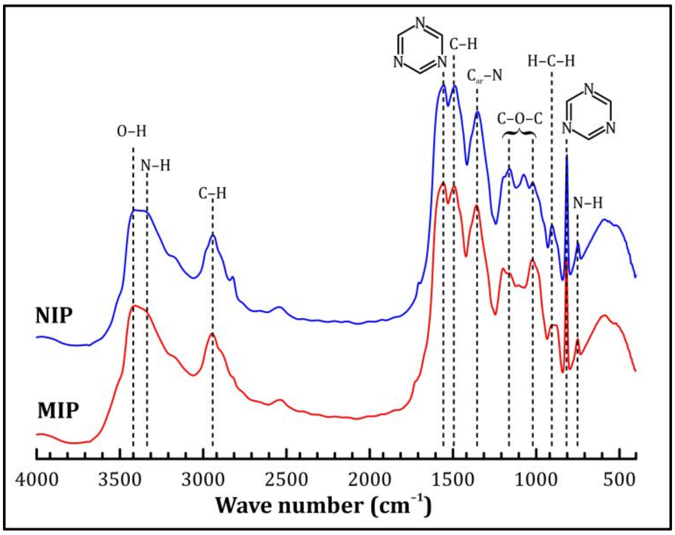

Figure S10. FT-IR spectra of NIP and MIP monoliths.

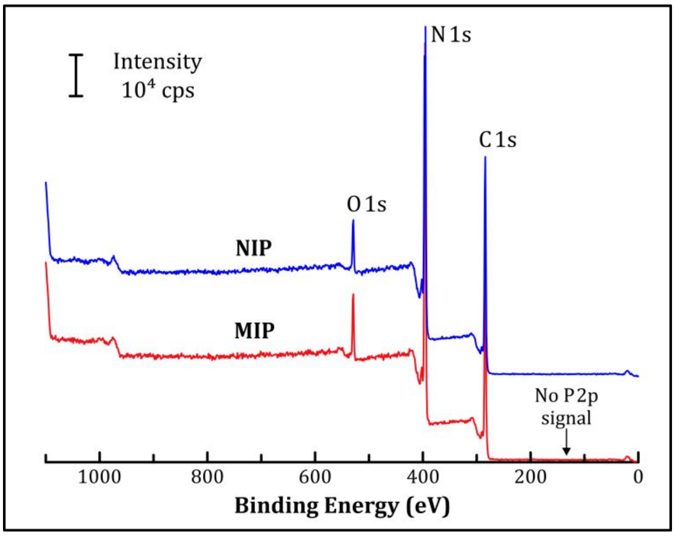

Figure S11. Survey X-ray photoelectron spectroscopic spectra of NIP and MIP monolith surfaces.

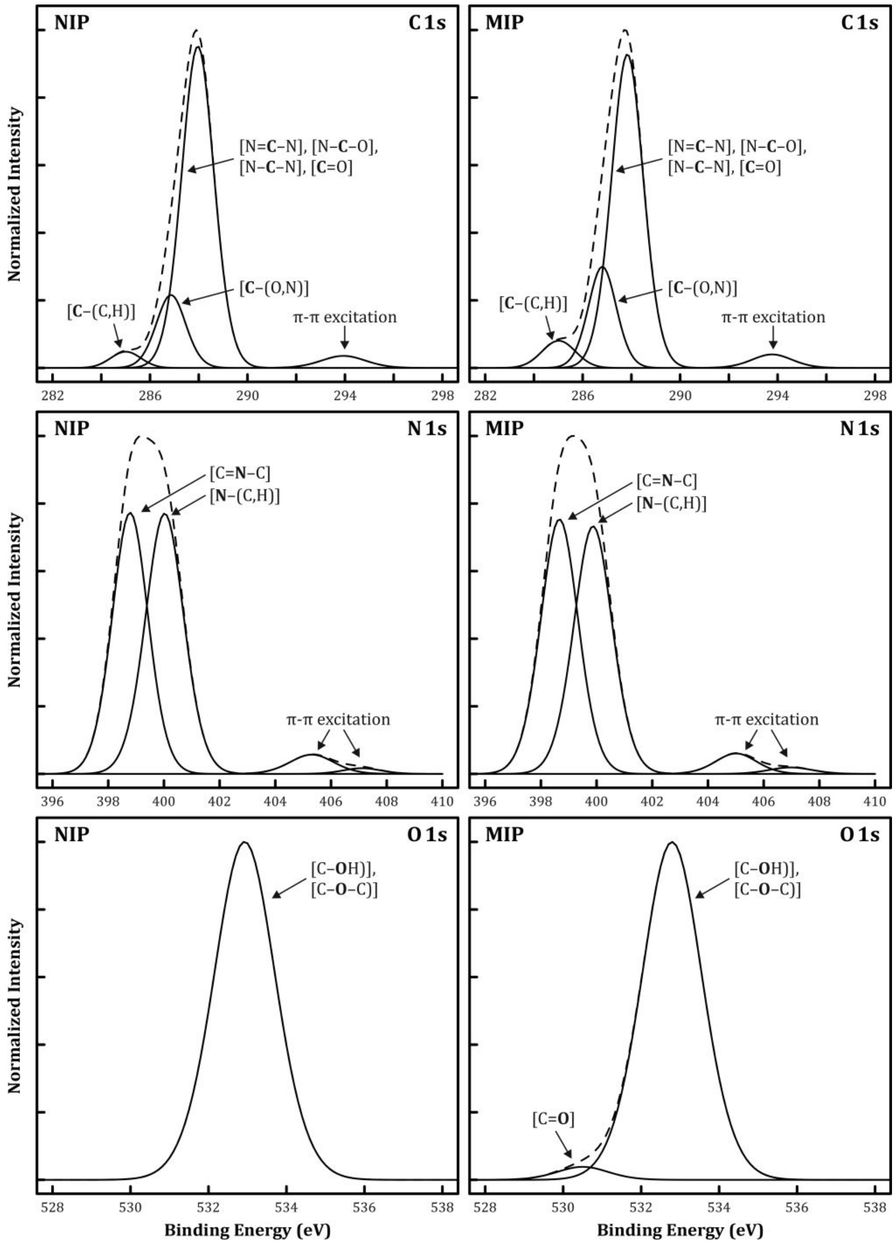

Figure S12. Representative high-resolution C 1s, N 1s, and O 1s XPS spectra of the non-imprinted polymer (NIP) and molecularly imprinted polymer (MIP). Dashed lines are the fitting envelopes.

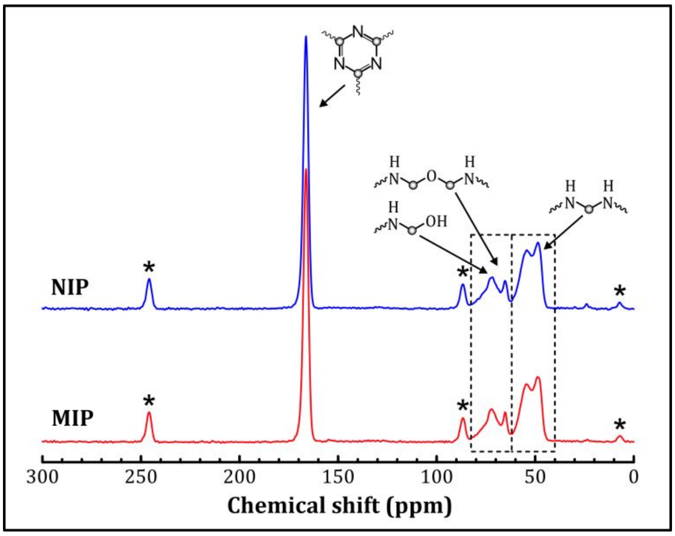

Figure S13. CP-MAS ^{13}C-NMR spectra of NIP and MIP monoliths. Asterisks denote rotational sidebands at 10 kHz.

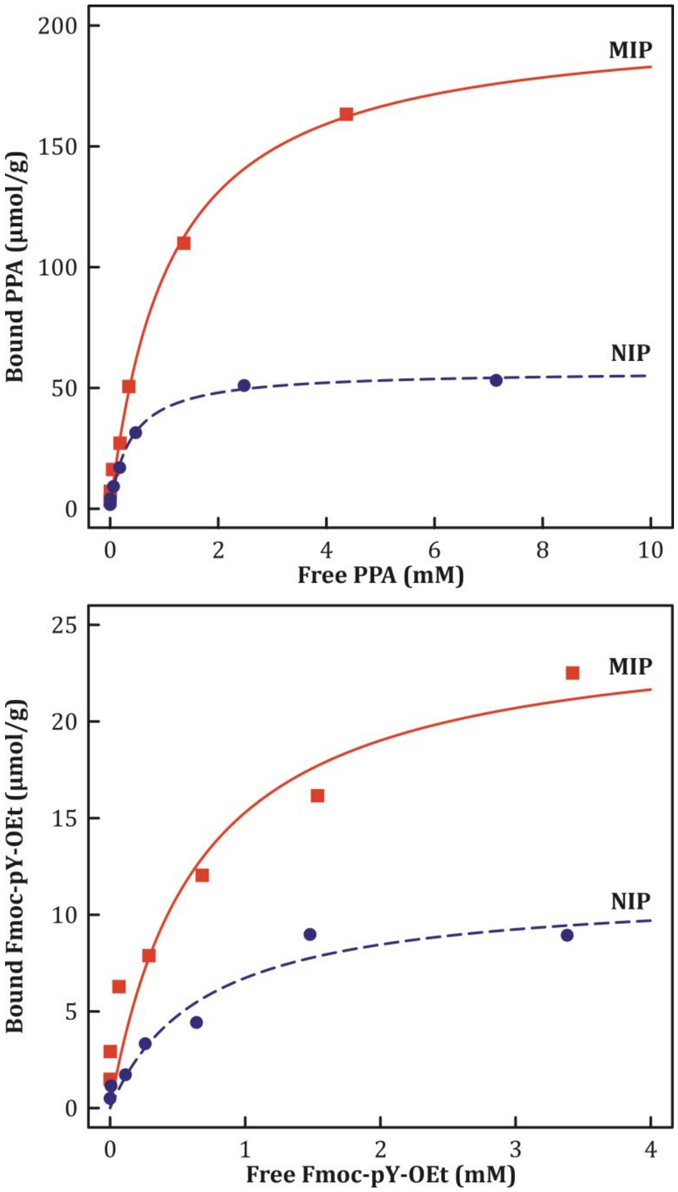

Figure S14. Binding curves showing the amount of bound versus free concentration for PPA and Fmoc pY OEt after incubation with NIP and MIP. The data were fitted to a Langmuir mono site model by non-linear regression. Initial concentrations were 0.1, 0.2, 0.5, 1, 2, 5, and 10 mM for PPA, and 0.05, 0.1, 0.25, 0.5, 1, 2.5, and 4 mM for Fmoc pY OEt.

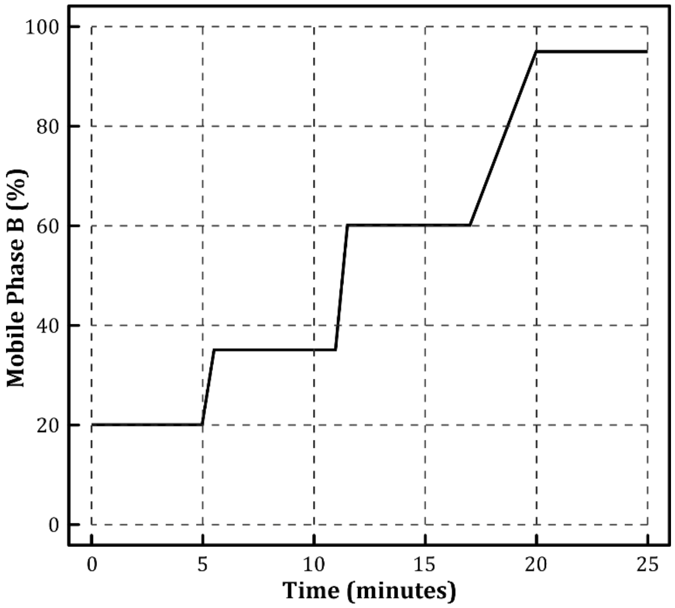

Figure S15. Step-gradient solvent program used in LC-MS evaluations of the capillary monolithic columns. Mobile phase A contained 0.1% formic acid in water. Mobile phase B was a mixture of 95% acetonitrile, 0.1% formic acid, and 4.9% water.

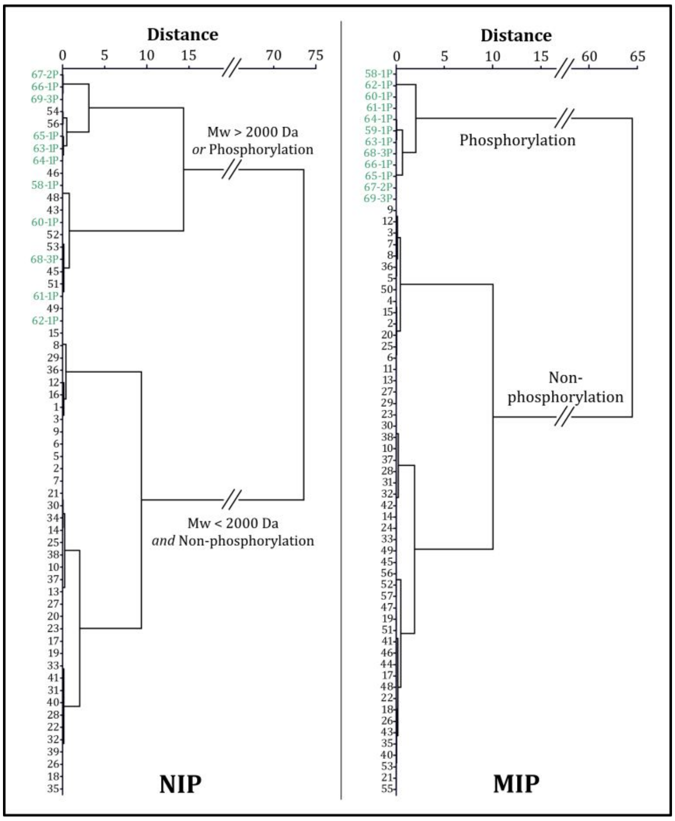

Figure S16. A hierarchical cluster analysis of the identified tryptic peptide on the NIP (left) and MIP (right) columns. The clusters to which these peptides are allocated are mainly dependent on their number of phosphorylations for the MIP column, and a combination of molecular weight and number of phosphorylations for NIP column.

Table S1. Properties of the Pluronics included in the initial synthesis attempts.

Pluronic	Composition[1)]	Molar mass[1)]	HLB[2)]
L61	$EO_2PO_{30}EO_2$	1,940	3
L121	$EO_5PO_{68}EO_5$	4,440	1
P123	$EO_{19}PO_{68}EO_{19}$	5,710	8
F127	$EO_{106}PO_{68}EO_{106}$	13,330	22

1) EO = ethylene oxide; PO = propylene oxide. Compositions according to ref S9; these vary slightly with information sources but this is the seminal paper by the inventor of the Pluronics. 2) Hydrophilic-lipophilic balance[S10] according to manufacturer product literature on the Internet, dated 2002.

Table S2. Surface elemental composition of MF monoliths by X-ray photoelectron spectroscopy.

Peak	Binding Energy (eV)		Atom (%)	
	NIP	MIP	NIP	MIP
C 1s [**C**–(C,H)]	285.0	285.0	1.66	2.96
C 1s [**C**–(O,N)]	286.8	286.8	7.40	9.35
C 1s [N=**C**–N, N–**C**–O, N–**C**–N, **C**=O]	287.9	287.8	35.73	33.01
C 1s [π-π* excitation]	293.9	293.8	1.82	1.78
O 1s [C=**O**]	N/D	530.5	N/D	0.14
O 1s [(C–**O**H), (C–**O**–C)]	532.9	532.8	3.40	4.09
N 1s [C=**N**–C]	398.8	398.6	22.99	23.04
N 1s [**N**–(C,H)]	400.0	399.9	24.40	22.93
N 1s [π-π* excitation]	405.3	405.0	2.07	2.02
N 1s [π-π* excitation]	407.1	407.0	0.53	0.67
Uncorrected C:N ratio[a)]	N/A	N/A	0.93:1	0.97:1
C:N ratio corrected for **C**–(C,H)[b)]	N/A	N/A	0.90:1	0.91:1
[**N**–(C,H)] : [C=**N**–C][c)]	N/A	N/A	1.06:1	1.00:1
[(C–**O**H), (C–**O**–C)] : [**N**–(C,H)][d)]	N/A	N/A	0.14:1	0.18:1

a) Ratio of the sum of all C 1s signals to the sum of all N 1s signals; b) Ratio of the sum of all C 1s signals less [**C**–(C,H)] to the sum of all N 1s signals; c) The ratio of amino/imino groups to melamine ring nitrogens; d) The ratio of residual ether bridges and unreacted methylol groups to melamine amino groups. N/A not applicable. N/D not detected.

Table S3. Langmuir monosite fitting of NIP and MIP with PPA and Fmoc-**pY**-OEt as probes.

	Phenylphosphoric acid (PPA)		Fmoc-pY-OEt	
	NIP	MIP	NIP	MIP
R-squared	0.9906	0.9926	0.9323	0.8902
B_{max} (μmol/m^2)	57.1 ± 2.0	203 ± 11	11.4 ± 1.6	25.1 ± 4.1
K_{eq} (mM^{-1})	2.64 ± 0.36	0.91 ± 0.13	1.45 ± 0.57	1.56 ± 0.76
Imprinting factor	N/A	3.56	N/A	2.20

N/A, Not applicable.

Table S4. List of 70 peptides, detected by LC-MS, extracted by msAccess (ProteoWizard), and identified by means of the SwissProt database (https://www.expasy.org).

ID	Peptide [1)]	Retention time [2)]	
		NIP column	MIP column
1	YNSYATALK	1.743	N/A
2	SLLANAEGDR	1.873	3.222
3	CELAAAMKR[1CAM]	1.967	3.225
4	GTDVQAWIR[1CAM]	N/A	3.015
5	LVHANFGTKK	1.701	2.966
6	AIIFYESNGK	1.713	3.025
7	LVNELTEFAK	1.808	3.082
8	ECCDKPLLEK	1.758	3.023
9	VLVLDTDYKK	1.736	2.966
10	TPEVDDEALEK	2.730	3.609
11	FKDLGEEHFK	N/A	3.577
12	YLGYLEQLLR	1.815	3.149
13	LFKSHPETLEK	1.826	3.093
14	GENQSPIELNTK	1.826	3.093
15	GTDVQAWIRGCR	1.790	3.025
16	FFVAPFPEVFGK	1.824	N/A
17	TDSTCRMVTSESK	2.077	3.372
18	LTTDFGNAEKTDR	1.938	3.193
19	ETYGDMADCCEK[2CAM]	3.419	4.060
20	IKAWTHFHFPGR	1.651	2.985
21	YPNCAYKTTQANK	2.566	3.379
22	EYEATLEECCAK[2CAM]	2.190	3.354
23	VPQVSTPTLVEVSR	1.782	3.069
24	HPGDFGADAQGAMNK	N/A	3.186
25	LKECCDKPLLEK[2CAM]	1.625	2.966
26	AFKDEDTQAMPFR	1.958	3.292
27	FSFLRDWVNHVR	1.958	3.284
28	LKPDPNTLCDEFK[1CAM]	1.958	3.284
29	VLPVPQKAVPYPQR	1.808	3.122
30	DSAQNSVIIVDKNGR	1.842	3.128
31	TPEVDDEALEKFDK	2.119	3.323
32	ENLCSTFCKEVVR[2CAM]	4.800	4.626
33	LSEKLDSTDFTGTIK	1.758	3.015
34	MLEERDLNVAIYDK	1.866	N/A
35	LSFNPTQLEEQCHI[1CAM]	1.747	3.059
36	LLPLDFGRYGDVVLR	1.751	3.122
37	HIATNAVLFFGRCVSP[1CAM]	2.033	3.295
38	YVRANGTTVLVGMPAGAK	1.816	3.097
39	VNMTDNVRPVQSLGQR	1.851	N/A
40	RPCFSALTPDETYVPK[1CAM]	1.930	3.264
41	NMAINPSKENLCSTFCK	2.220	3.354
42	RDPSLKPLSVSYNPATAK	N/A	5.844
43	LKPDPNTLCDEFKADEK[1CAM]	1.935	3.250
44	EGDSSVANSGLAALITDGPGGAK	N/A	3.599

**45**	MVNNGHSFNVEYDDSQDK	3.382	3.874
**46**	DILNQITKPNDVYSFSLASR	1.620	2.954
**47**	LAVDEEENADNNTKANVTKPK	N/A	2.991
**48**	ATDGGAHGVINVSVSEAAIEASTR	2.492	3.282
**49**	ECCDKPLLEKSHCIAEVEK[3CAM]	1.876	3.052
**50**	IPIKYLEFISEAIIHVLHSR	N/A	3.100
**51**	CKPVNTFVHESLADVQAVCSQK[1CAM]	4.841	5.592
**52**	MVNNGHSFNVEYDDSQDKAVLK	2.151	3.386
**53**	QFHFHWGITDDCGSEHLVDGAK[1CAM]	4.950	4.608
**54**	LVQFHFHWGSSDDQGSEHTVDR	4.919	N/A
**55**	LKGPVAPQEWQGSLLGSPYHLGPGPR	1.967	3.212
**56**	CKPVNTFVHESLADVQAVCSQKNVACK[2CAM]	N/A	3.323
**57**	FRTLNFNAEGEPELLMLANWRPAQPLK	N/A	3.305
**58-1P**	SIGGEVFIDFTK[1P]	5.086	5.724
**59-1P**	TVDMESTEVFTK[1P]	N/A	7.320
**60-1P**	TVMENFVAFVDK[1P]	2.215	3.195
**61-1P**	SIGGEVFIDFTKEK[1P]	3.225	3.670
**62-1P**	VPQLEIVPNSAEER[1P]	5.352	6.325
**63-1P**	EELFTSLGGEVFIDFTKEK[1P]	4.950	4.608
**64-1P**	CCSDVFNQVVKSISIVGSYVGNR[1P]	4.910	5.032
**65-1P**	MMDQARSAFSNLFGGEPLSYTR[1P]	4.919	8.094
**66-1P**	ELEELNVPGEIVESLSSSEESITR[1P]	4.009	3.707
**67-2P**	RELEELNVPGEIVESLSSSEESITR[2P]	2.204	3.224
**68-3P**	SAFSNLFGGEPLSYTR[3P]	2.011	3.292
**69-3P**	ELEELNVPGEIVESLSSSEESITR[3P]	2.052	3.348
**70-3P**	GENQSPIELNTKEISHDPSLKPWTASYDPGSAK[3P]	4.950	8.139

1) [CAM] = alkylated cysteine; [P] = phosphorylated serine or threonine. 2) N/A: Not found or peak intensity is lower than 10^5.

References

(S1) Brunauer, S.; Emmett, P. H.; Teller, E. Adsorption of Gases in Multimolecular Layers. *J. Am. Chem. Soc.* **1938**, *60*, 309–319.

(S2) Barrett, E. P.; Joyner, L. G.; Halenda, P. P. The Determination of Pore Volume and Area Distributions in Porous Substances. I. Computations from Nitrogen Isotherms. *J. Am. Chem. Soc.* **1951**, *73*, 373–380.

(S3) Fung, B. M.; Khitrin, A. K.; Ermolaev, K. An improved broadband decoupling sequence for liquid crystals and solids. *J. Magn. Reson.* **2000**, *142*, 97-101.

(S4) Koley, S.; Ghosh, S. A deeper insight into an intriguing acetonitrile–water binary mixture: synergistic effect, dynamic Stokes shift, fluorescence correlation spectroscopy, and NMR studies. *Phys. Chem. Chem. Phys.* **2016**, *18*, 32308–32318.

(S5) Chambers, M. C.; MacLean, B.; Burke, R.; Amode, D.; Ruderman, D. L.; Neumann, S.; Gatto, L.; Fischer, B.; Pratt, B.; Egertson, J.; Hoff, K.; Kessner, D.; Tasman, N.; Shulman, N.; Frewen, B.; Baker, T. A.; Brusniak, M.-Y.; Paulse, C.; Creasy, D.; Flashner, L.; Kani, K.; Moulding, C.; Seymour, S. L.; Nuwaysir, L. M.; Lefebvre, B.; Kuhlmann, F.; Roark, J.; Rainer, P.; Detlev, S.; Hemenway, T.; Huhmer, A.; Langridge, J.; Connolly, B.; Chadick, T.; Holly, K.; Eckels, J.; Deutsch, E. W.; Moritz, R. L.; Katz, J.E.; Agus, D. B.; MacCoss, M.; Tabb, D. L.; Mallick, P. A cross-platform toolkit for mass spectrometry and proteomics *Nat. Biotechnol.* **2012**, *30*, 918–920; URL: http://proteowizard.sourceforge.net

(S6) signal developers, signal: Signal processing, **2013**. URL: http://r-forge.r-project.org/projects/signal

(S7) R Core Team, R: A language and environment for statistical computing, R Foundation for Statistical Computing: Vienna, Austria, **2020**. URL: https://www.R-project.org

(S8) Ikai, A.J. Thermostability and Aliphatic Index of Globular Proteins. *J. Biochem.* **1980**, *88*, 1895–1898.

(S9) Schmolka, I. K. Artificial skin. I. Preparation and properties of pluronic F-127 gels for treatment of burns. *J. Biomed. Mater. Res.* **1972**, *6*, 571-582.

(S10) a) Griffin, W. C. Classification of Surface-Active Agents by "HLB". *J. Soc. Cosmet. Chem.*, **1949**, *1*, 311–326; b) Griffin, W. C. Calculation of HLB values of non-ionic Surfactants. *J. Soc. Cosmet. Chem.*, **1954**, *5*, 249–256.
